# Genome-wide studies reveal factors associated with circulating uromodulin and its relationships to complex diseases

**DOI:** 10.1172/jci.insight.157035

**Published:** 2022-05-23

**Authors:** Yong Li, Yurong Cheng, Francesco Consolato, Guglielmo Schiano, Michael R. Chong, Maik Pietzner, Ngoc Quynh H. Nguyen, Nora Scherer, Mary L. Biggs, Marcus E. Kleber, Stefan Haug, Burulça Göçmen, Marie Pigeyre, Peggy Sekula, Inga Steinbrenner, Pascal Schlosser, Christina B. Joseph, Jennifer A. Brody, Morgan E. Grams, Caroline Hayward, Ulla T. Schultheiss, Bernhard K. Krämer, Florian Kronenberg, Annette Peters, Jochen Seissler, Dominik Steubl, Cornelia Then, Matthias Wuttke, Winfried März, Kai-Uwe Eckardt, Christian Gieger, Eric Boerwinkle, Bruce M. Psaty, Josef Coresh, Peter J. Oefner, Guillaume Pare, Claudia Langenberg, Jürgen E. Scherberich, Bing Yu, Shreeram Akilesh, Olivier Devuyst, Luca Rampoldi, Anna Köttgen

**Affiliations:** 1 Institute of Genetic Epidemiology, Faculty of Medicine and Medical Center, and; 2 Faculty of Biology, University of Freiburg, Freiburg, Germany.; 3 Molecular Genetics of Renal Disorders group, Division of Genetics and Cell Biology, IRCCS Ospedale San Raffaele, Milan, Italy.; 4 Institute of Physiology, University of Zurich, Zurich, Switzerland.; 5 Population Health Research Institute and Thrombosis and Atherosclerosis Research Institute, David Braley Cardiac, Vascular and Stroke Research Institute, Hamilton Health Sciences, Hamilton, Ontario, Canada.; 6 Department of Biochemistry and Biomedical Sciences and; 7 Department of Pathology and Molecular Medicine, Faculty of Health Science, McMaster University, Hamilton, Ontario, Canada.; 8 Medical Research Council (MRC) Epidemiology Unit, Institute of Metabolic Science, University of Cambridge School of Clinical Medicine, Cambridge, United Kingdom.; 9 Computational Medicine, Berlin Institute of Health at Charité – Universitätsmedizin Berlin, Berlin, Germany.; 10 Department of Epidemiology, Human Genetics and Environmental Sciences, School of Public Health, University of Texas Health Science Center at Houston, Houston, Texas, USA.; 11 Spemann Graduate School of Biology and Medicine, University of Freiburg, Freiburg, Germany.; 12 Cardiovascular Health Research Unit, Department of Medicine, and; 13 Department of Biostatistics, University of Washington, Seattle, Washington, USA.; 14 SYNLAB MVZ Humangenetik Mannheim GmbH, Mannheim, Germany.; 15 Vth Department of Medicine, Medical Faculty Mannheim, Heidelberg University, Mannheim, Germany.; 16 Department of Medicine, Michael G. DeGroote School of Medicine, McMaster University, Hamilton, Ontario, Canada.; 17 Department of Epidemiology, Johns Hopkins Bloomberg School of Public Health, Baltimore, Maryland, USA.; 18 MRC Human Genetics Unit, Institute of Genetics and Cancer, University of Edinburgh, Western General Hospital, Edinburgh, United Kingdom.; 19 Division of Nephrology, School of Medicine, Johns Hopkins University, Baltimore, Maryland, USA.; 20 Department of Medicine IV: Nephrology and Primary Care, Faculty of Medicine and Medical Center, University of Freiburg, Freiburg, Germany.; 21 Institute of Genetic Epidemiology, Medical University of Innsbruck, Innsbruck, Austria.; 22 Institute of Epidemiology, Helmholtz Center Munich, German Research Center for Environmental Health, Neuherberg, Germany.; 23 Chair of Epidemiology, Institute for Medical Information Processing, Biometry, and Epidemiology, Faculty of Medicine, Ludwig-Maximilians-Universität (LMU), Munich, Germany.; 24 Medical Clinic and Policlinic IV, Hospital of the University of Munich, LMU Munich, Munich, Germany.; 25 Division of Nephrology, Tufts Medical Center, Boston, Massachusetts, USA.; 26 Department of Nephrology, Klinikum rechts der Isar, Technical University Munich, Munich, Germany.; 27 Boehringer Ingelheim International GmbH, Ingelheim, Germany.; 28 Clinical Institute of Medical and Chemical Laboratory Diagnostics, Medical University of Graz, Graz, Austria.; 29 SYNLAB Academy, SYNLAB Holding Deutschland GmbH, Augsburg and Mannheim, Germany.; 30 Department of Nephrology and Medical Intensive Care, Charité – Universitätsmedizin Berlin, Berlin, Germany.; 31 Department of Nephrology and Hypertension, University Hospital Erlangen, Friedrich-Alexander-Universität Erlangen-Nürnberg, Erlangen, Germany.; 32 Research Unit of Molecular Epidemiology, Helmholtz Center Munich, German Research Center for Environmental Health, Neuherberg, Germany.; 33 German Center for Diabetes Research (DZD), Partner Munich, Neuherberg, Germany.; 34 Human Genetics Center, School of Public Health, University of Texas Health Science Center at Houston, Houston, Texas, USA.; 35 Human Genome Sequencing Center, Baylor College of Medicine, Houston, Texas, USA.; 36 Department of Epidemiology and; 37 Department of Health Systems and Population Health, School of Public Health, University of Washington, Seattle, Washington, USA.; 38 Institute of Functional Genomics, University of Regensburg, Regensburg, Germany.; 39 Munich Clinic Harlaching, LMU Munich, Munich, Germany.; 40 Department of Laboratory Medicine and Pathology, University of Washington, Seattle, Washington, USA.; 41 Centre for Integrative Biological Signalling Studies (CIBSS), University of Freiburg, Freiburg, Germany.

**Keywords:** Genetics, Nephrology, Chronic kidney disease, Population genetics

## Abstract

Uromodulin (*UMOD*) is a major risk gene for monogenic and complex forms of kidney disease. The encoded kidney-specific protein uromodulin is highly abundant in urine and related to chronic kidney disease, hypertension, and pathogen defense. To gain insights into potential systemic roles, we performed genome-wide screens of circulating uromodulin using complementary antibody-based and aptamer-based assays. We detected 3 and 10 distinct significant loci, respectively. Integration of antibody-based results at the *UMOD* locus with functional genomics data (RNA-Seq, ATAC-Seq, Hi-C) of primary human kidney tissue highlighted an upstream variant with differential accessibility and transcription in uromodulin-synthesizing kidney cells as underlying the observed *cis* effect. Shared association patterns with complex traits, including chronic kidney disease and blood pressure, placed the *PRKAG2* locus in the same pathway as *UMOD*. Experimental validation of the third antibody-based locus, *B4GALNT2*, showed that the p.Cys466Arg variant of the encoded N-acetylgalactosaminyltransferase had a loss-of-function effect leading to higher serum uromodulin levels. Aptamer-based results pointed to enzymes writing glycan marks present on uromodulin and to their receptors in the circulation, suggesting that this assay permits investigating uromodulin’s complex glycosylation rather than its quantitative levels. Overall, our study provides insights into circulating uromodulin and its emerging functions.

## Graphical abstract

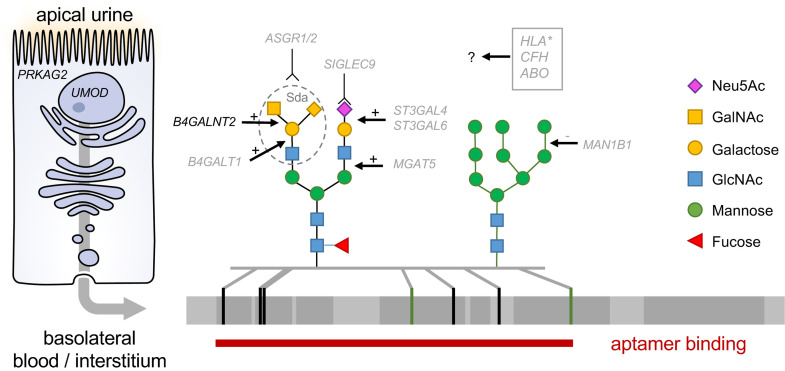

## Introduction

Chronic kidney disease (CKD) can progress to kidney failure, is a major risk factor for cardiovascular morbidity and mortality, and is a leading cause of death (1–3). CKD affects approximately 10% of adults (1). Genome-wide association studies (GWAS) of kidney function, CKD, and CKD progression in population-based studies have consistently identified the largest effect for common variants at the uromodulin (*UMOD*) locus (4–7). The encoded protein uromodulin, previously named Tamm-Horsfall protein, is the most abundant protein in the urine of healthy individuals (8). It is exclusively synthesized in the kidney’s thick ascending limb (TAL) of the loop of Henle (LOH) and the distal convoluted tubule (DCT) (9). Urinary uromodulin has important roles in protecting against urinary tract infections (10). Glycosylation accounts for approximately 30% of the mature protein’s molecular weight in urine and may be important for some of the protein’s functions, including an emerging immunomodulatory role (8).

Common CKD risk variants in *UMOD* are also associated with higher risk of hypertension, hyperuricemia, and gout and lower risk of kidney stone disease (4, 11–14). Their association with higher uromodulin transcript levels in kidney (7, 15) and higher uromodulin levels in urine (7, 16) directly implicates a pathophysiologic role of uromodulin. Rare mutations in *UMOD* cause one of the most common monogenic kidney diseases, autosomal-dominant tubulointerstitial kidney disease (17, 18).

*UMOD* is hence a main driver of genetic kidney disease, and genetic studies of the kidney-specific protein uromodulin may yield insights not only into kidney disease but also into the protein’s other diverse functions and associated diseases. Such studies can also reveal regulators and interaction partners that can help to understand potential consequences of therapeutic manipulation and may reveal new entry points to do so, with the final goal to reach pharmacological intervention (19). Previous studies of uromodulin have almost exclusively focused on urine. The protein is, however, also released from the basolateral membrane of renal TAL and DCT cells and reaches the blood, where its concentration is about 100-fold lower than in urine (8). In a previous study, urine and plasma uromodulin levels were moderately correlated (20), though they are both associated with the kidney function measure estimated glomerular filtration rate (eGFR). The mechanisms influencing circulating uromodulin, whether circulating and urine uromodulin share association patterns with complex diseases, and any factors related to the glycans carried by uromodulin are unknown. Quantification of circulating uromodulin on a population scale has recently become feasible (21–24). A small GWAS of serum uromodulin levels reported only an association with the known CKD-associated *UMOD* variants in *cis* (23).

Here, we performed meta-analyses of GWAS of circulating uromodulin to obtain insights into factors that may be relevant to CKD pathophysiology and into any systemic functions of this kidney-specific protein. Using an antibody-based assay, we (i) identified an upstream variant at the *UMOD* locus with differential accessibility and transcription in human uromodulin-synthesizing kidney cell types and compartments that was strongly associated with circulating and urine uromodulin, CKD, and hypertension; (ii) placed the *PRKAG2* locus in the same pathway as *UMOD* with respect to its disease associations; and (iii) showed that p.Cys466Arg in the uromodulin-glycosylating enzyme B4GALNT2 was a loss-of-function allele leading to higher serum uromodulin levels. Using an aptamer-based assay, we identified non-overlapping loci that pointed to enzymes writing glycan marks present on uromodulin and to their receptors in the circulation. Together, our study based on human genetic evidence provides insights into circulating uromodulin and its emerging functions.

## Results

### GWAS meta-analyses identify 13 genetic loci associated with circulating uromodulin.

Characteristics of the 32,055 individuals from 7 participating studies (Atherosclerosis Risk in Communities [ARIC], Cardiovascular Health Study [CHS], Fenland, German Chronic Kidney Disease [GCKD], Cooperative Health Research in the Region Augsburg [KORA], LUdwigshafen RIsk and Cardiovascular [LURIC], Outcome Reduction with an Initial Glargine Intervention [ORIGIN]), including distributions of age, sex, and eGFR, are shown in Supplemental Table 1; supplemental material available online with this article; https://doi.org/10.1172/jci.insight.157035DS1 There were 29,439 participants of European ancestry (EA), 400 African American (AA) participants, and 2216 Hispanic (HIS) participants. GWAS of age-, sex-, and eGFR-adjusted and rank-based inverse normal transformed circulating uromodulin measurements were carried out in each of the 7 studies using densely imputed genotypes (25, 26) (Supplemental Table 2) and combined via meta-analysis (Methods).

**Figure 1:**
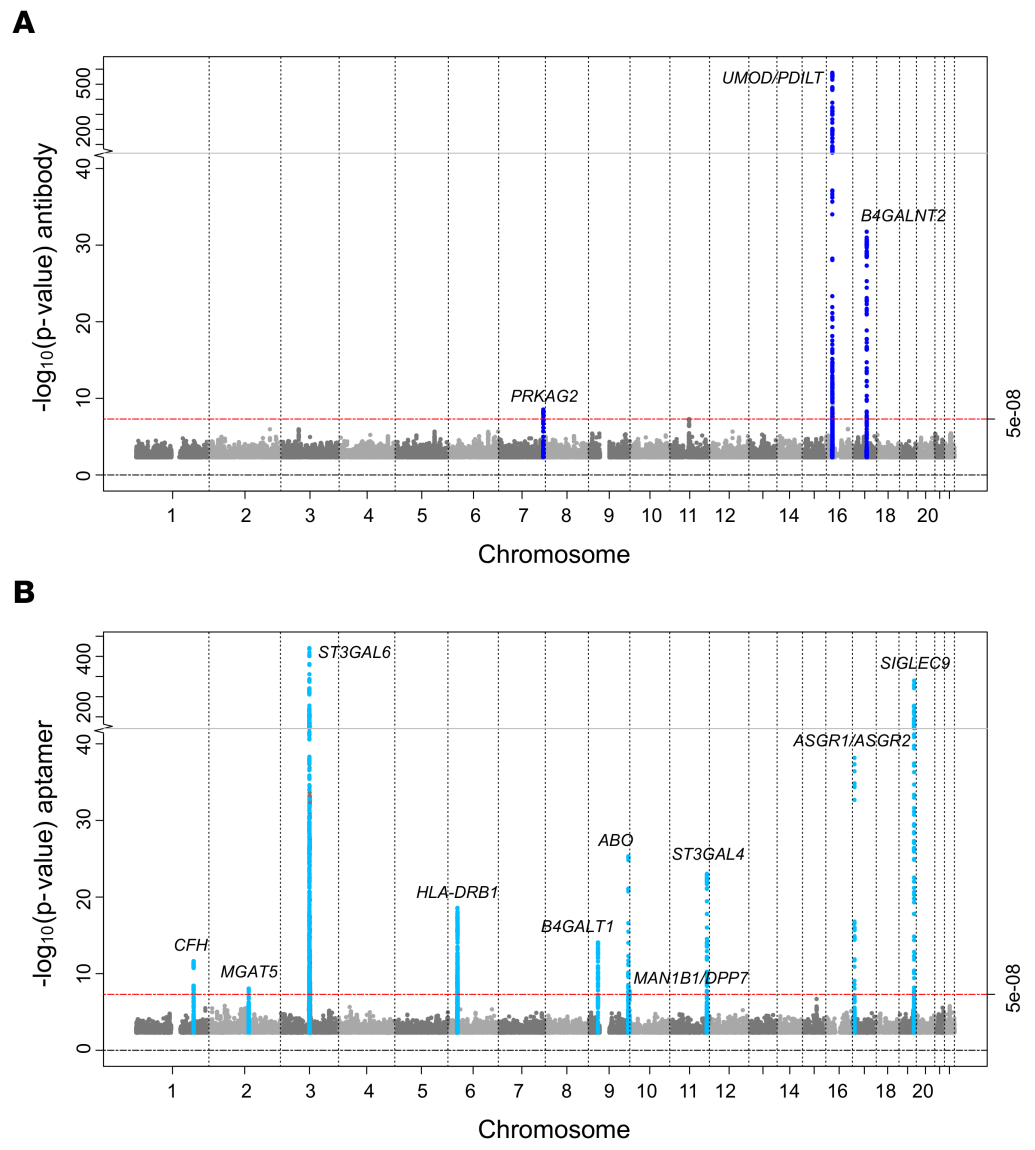
Manhattan plot of GWAS of antibody-based and aptamer-based circulating uromodulin. The plots represent, for each SNP, the *P* value from meta-analyses of GWAS of antibody-based (*n* = 13,985, dark blue, **A**) and of aptamer-based circulating uromodulin (*n* = 18,070, light blue, **B**). The *x* axis shows chromosomal location and the *y* axis the –log_10_(*P* value) of SNP associations with circulating uromodulin. The plots were generated using the R package EasyStrata v8.6. Meta-analyses of X chromosomal markers did not yield any significant findings.

Trans-ethnic meta-analysis of 10,735,251 genetic variants of minor allele frequency (MAF) more than 1% across 5 studies with antibody-based uromodulin quantification (CHS, GCKD, KORA, LURIC, ORIGIN; *n* = 13,985) revealed 3 genomic loci with at least 1 significantly associated (*P* < 5 × 10^–8^) genetic variant (Figure 1A and Supplemental Table 3): *UMOD*/*PDILT* (index SNP rs77924615, *P* = 6.4 × 10^–577^), *B4GALNT2* (rs7224888, *P* = 1.8 × 10^–32^), and *PRKAG2* (rs55791829, *P* = 2.9 × 10^–9^). The genomic control parameter was 0.99, consistent with the absence of undetected population stratification (Supplemental Figure 1A). The estimated SNP-based heritability of uromodulin was 0.135 (95% confidence interval [CI] 0.010–0.259, Methods). Except for the *UMOD* locus, there was little heterogeneity of genetic effects in the 5 contributing studies (Supplemental Figure 2). The index variant rs77924615 at the locus with the strongest association, *UMOD*/*PDILT*, explained an estimated 18% of the serum uromodulin variance (Table 1, Methods).

The GWAS meta-analysis of 8,815,558 genetic variants across 2 studies with plasma aptamer uromodulin readout (ARIC and Fenland; *n* = 18,070) showed no evidence of inflation (λ = 0.99; Supplemental Figure 1B) and revealed 10 genome-wide significant loci (Figure 1B and Supplemental Table 3), with the statistically strongest association observed at rs34211178 upstream of *ST3GAL6* on chromosome 3 (*P* = 6.9 × 10^–442^). *ST3GAL6* codes for ST3 beta-galactoside alpha-2,3-sialyltransferase (27), an enzyme with alpha-2,3-sialyltransferase activity toward Gal-beta1,4-GlcNAc structures that are present on the glycoprotein uromodulin (28). The largest effect size was observed for a low-frequency variant in asialoglycoprotein receptor 2 (*ASGR2*), with each minor allele associated with 1 standard deviation higher age- and sex-adjusted plasma aptamer uromodulin readout. The estimated SNP-based heritability was 0.177 (95% CI –0.032–0.386). For each of the 2 assays, regional association plots for all 13 loci that achieved genome-wide significance are shown in Supplemental Figure 3, with association statistics in Supplemental Table 3.

The 2 meta-analyses of antibody- and aptamer-based uromodulin measurements identified different genetic loci. A *cis* association between SNPs in the *UMOD* gene and levels of the encoded protein uromodulin were only observed with antibody quantification, supporting that this assay measures the amount of protein. Within each of the 2 meta-analyses, the association results showed consistent effect sizes and directions in all contributing studies, and both assays had low coefficients of variation (Methods). Moreover, genes identified with both assays can be connected to uromodulin through different sources of external evidence (see below). This indicates that the antibody- and aptamer-based assays for circulating uromodulin deliver reproducible measurements but assess different properties of their respective targets, such as protein amount and glycosylation pattern, respectively.

### Secondary analyses, sex-specific effects, and association with urine uromodulin levels.

Genome-wide discovery screens without adjustment for eGFR showed virtually identical results (Supplemental Figure 4, A and B), indicating that kidney function did not confound genetic associations with uromodulin. A secondary analysis restricted to 11,369 EA participants with antibody-based measurements yielded very similar results as the primary trans-ethnic meta-analysis (Supplemental Table 3).

We next evaluated the presence of sex-specific genetic effects, motivated by the observation that women have higher serum uromodulin levels than men (22, 29, 30). Higher circulating uromodulin in women as compared with men was observed for both the antibody- (mean of the mean uromodulin 103.62 ng/mL in women versus 92.76 ng/mL in men) and the aptamer-based assay (10,329 versus 9813 relative fluorescence units). Sex-specific analyses identified several genome-wide significant loci for both assays (Supplemental Figure 5), all of which were also identified in the primary combined analyses. The index SNPs at the 13 significant loci did not show evidence for sex-specific differences (Supplemental Figure 6), nor did GWAS of the X chromosome or a genome-wide test for differences of SNP effects on uromodulin between men and women yield significant findings (Supplemental Table 4).

Given that a previous GWAS meta-analysis of urine uromodulin reported significant associations at the *UMOD* locus (16), we queried the association between the 13 index SNPs identified in this study and urine uromodulin levels among 29,262 EA individuals (Methods). Except for rs77924615 at *UMOD*/*PDILT* (*P* = 5.3 × 10^–97^), which explained 1.4% of the urine uromodulin variance, none of the other SNPs showed significant (*P* < 3.8 × 10^–3^ = 0.05/13) associations (Supplemental Table 5).

### Prioritization of causal variants in uromodulin-associated loci.

Statistical fine mapping was carried out to identify the most likely causal variants in uromodulin-associated loci (Methods). Conditional analyses supported the presence of more than 1 independent signal at *UMOD*/*PDILT* (*n* = 2), *B4GALNT2* (*n* = 3), *ST3GAL6* (*n* = 4), and *ASGR1*/*ASGR2* (*n* = 2; Supplemental Table 6). For each of 20 independent, uromodulin-associated signals within the 13 identified loci, we calculated a SNP set that contains the variant driving the respective association signal with 99% posterior probability. There were 12 sets with fewer than 20 variants, 5 of which had fewer than 5 variants (Supplemental Table 6).

**Figure 2:**
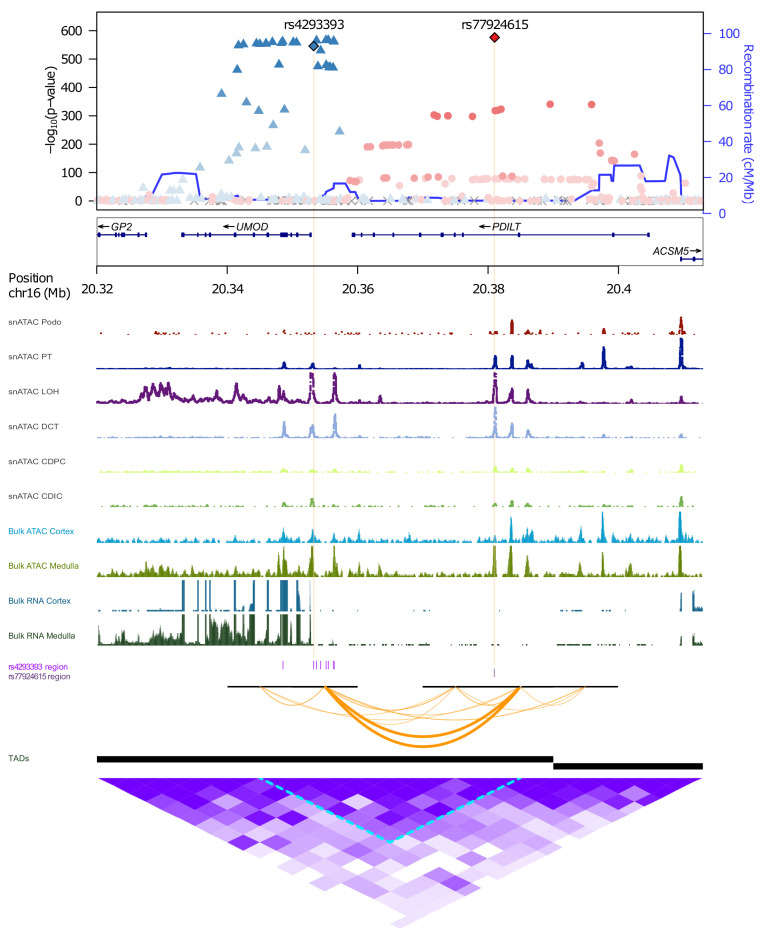
Functional genomic annotation of significantly associated independent variants at the *UMOD*/*PDILT* locus using gene expression and chromatin accessibility data from primary human kidney. The upper part shows the regional association plot of the *UMOD/PDILT* locus, using the 2 independent variants as reference SNPs. For nonreference SNPs, the extent of linkage disequilibrium (LD) with the reference SNP with higher correlation is shown by color gradients. Genetic positions (*x* axis) represent GRCh38 coordinates. Open chromatin peaks in different kidney cell type tracks based on single nuclear (sn)ATAC-Seq are shown underneath the regional association plot. Gene expression and open chromatin tracks of cortex (light blue tracks) and medulla (dark green tracks) based on bulk RNA-Seq and ATAC-Seq are shown in the lower part as density peaks. SNPs in the 2 independent credible sets with posterior probability (PP) > 0.01 are marked by ticks (purple) and the 10 kb windows encompassing them by the black horizontal bars. Hi-C data generated from kidney cortex was analyzed for contacts (orange arcs) between the 10 kb windows encompassing the indicated SNPs with contacts closest to the causal SNPs arbitrarily shown in bold. Intervals for DomainCaller computed topology associated domains (TADs) are shown as black bars below contact arcs. A heatmap of all Hi-C contacts encompassing this region is shown at the bottom (purple). Podo, podocyte; PT, proximal tubule; LOH, loop of Henle; DCT, distal convoluted tubule; CDPC, collecting duct principal cells; CDIC, collecting duct intercalated cells.

Credible set variant annotation showed several noteworthy findings (Supplemental Table 7). The antibody-based association on chromosome 16 could be mapped to a single intronic variant, rs77924615, in *PDILT*, the gene upstream of *UMOD*. Another independent set of 8 variants in the locus mapped to *UMOD* (Figure 2), with the lead SNP rs4293393 experimentally shown to affect *UMOD* transcription (15). To study whether rs77924615 may be an upstream variant regulating *UMOD* transcription, we generated functional genomic annotation data of chromatin accessibility (ATAC-Seq) and gene expression (RNA-Seq) from cortex and medulla of native human kidney tissue (Methods) that showed transcription of *UMOD*, more strongly in medulla than in cortex, but not of *PDILT*. Both independent variants, rs77924615 in *PDILT* and rs4293393 in the *UMOD* promoter, mapped into regions of open chromatin in medulla, where *UMOD* transcript levels in human kidney are highest (GTEx Project V8) (31). These regions aligned with open chromatin in LOH and DCT kidney cells from publicly available single-nucleus ATAC-Seq data (Methods), which was not observed in several other kidney and immune cell types (Figure 2). Both variants mapped into the same topological associated domain, with predicted contacts based on chromatin conformation capture (Hi-C, Methods). Thus, the identified SNPs likely are regulatory variants in kidney cell types producing uromodulin.

The lead SNP at the *B4GALNT2* locus, rs7224888, was identified with the antibody assay and is a missense variant. Its minor C allele encodes a cysteine-to-arginine substitution (p.Cys466Arg; NP_703147.2) in the encoded enzyme beta-1,4-N-acetyl-galactosaminyltransferase 2. The C allele was associated with higher circulating uromodulin in our study and has been linked to the absence of the Sda antigen (32), a blood group antigen synthesized by B4GALNT2 that is present on uromodulin (33). The *B4GALNT2* locus also contained an independent small credible set of 3 variants. The most likely causal rs72835417 mapped into a splice region; its minor allele was associated with higher uromodulin in our study, and with lower *B4GALNT2* expression (*P* = 1.8 × 10^–7^) in micro-dissected kidney tubules (34), further supporting that reduced B4GALNT2 function relates to higher circulating uromodulin.

At sialic acid-binding Ig-like lectin-9 (*SIGLEC9*), the major G allele at the most likely causal variant rs2075803 leads to a lysine-to-glutamine substitution in SIGLEC9 (p.Lys100Glu; NP_001185487.1). It was associated with lower aptamer signal in our study (*P* = 3.8 × 10^–100^) and with lower circulating SIGLEC9 protein (*P* = 6 × 10^–2142^) (35) and serum C-reactive protein (*P* = 5 × 10^–10^) (36) in previous studies. The encoded sialic acid-binding Ig-like lectin-9 is an inhibitory receptor mainly present on neutrophils and monocytes. It has been experimentally shown to interact with urinary uromodulin (37), indicating that genetically encoded variation in *SIGLEC9* levels relates to differences in the aptamer readout of circulating uromodulin.

### Uromodulin-associated loci are associated with distinct sets of biomarkers and diseases.

**Figure 3:**
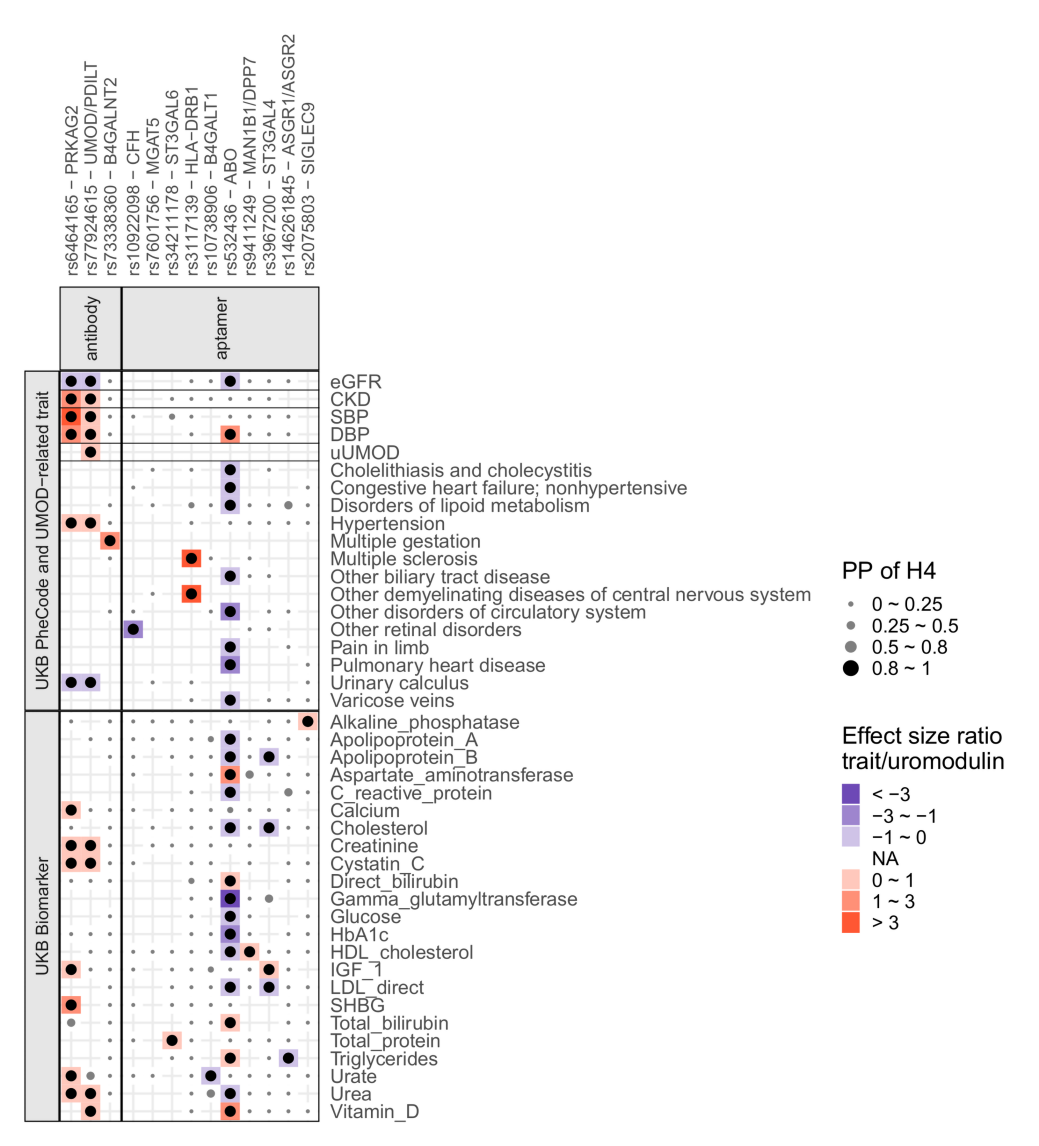
Summary of findings from colocalization of uromodulin signals with associations from GWAS of biomarkers and diseases. The colocalization analyses’ findings are shown in 2 categories, biomarkers and diseases. The *x* axis indicates the index SNPs with the likely causal genes. The *y* axis shows the traits for biomarkers and diseases, and only top-level UK Biobank PheCodes are shown. Within each category, horizontal lines separate different data sources (Methods). Included traits had at least 1 positive colocalization signal (PP of H4 > 0.8, Methods). Dots are black when PP of H4 > 0.8 and gray otherwise and scaled in size to reflect the different ranges of PP of H4. The trait-to-uromodulin effect size ratios are shown as gradient background for positive colocalization signals, with red indicating positive and blue negative changes per unit higher uromodulin levels. The colocalization with urine uromodulin (uUMOD) was based on conditional association statistics (Methods). H4, hypothesis that 1 shared SNP underlies the association with 2 traits; PP, posterior probability.

Genetic studies have linked variation in *UMOD* to monogenic autosomal-dominant tubulointerstitial kidney disease (17, 38), complex kidney function traits and CKD (4), blood pressure and hypertension (11, 12), uric acid levels and gout (13), as well as kidney stone disease (14). In order to investigate whether any of the 13 significant loci share phenotype association patterns, and to detect additional disease associations that may be mediated by altered uromodulin levels or properties, we performed colocalization with (i) levels of 30 biomarkers and (ii) 1404 complex traits and diseases based on data from the UK Biobank, as well as (iii) additional traits previously linked to common *UMOD* variants — namely eGFR, CKD, systolic blood pressure (SBP), diastolic blood pressure (DBP), and uromodulin levels in urine (Methods). Interestingly, genetic associations with antibody-based circulating uromodulin at the *PRKAG2* and *UMOD*/*PDILT* loci shared a very similar pattern of colocalization with numerous kidney-related traits (creatinine, cystatin C, urea, eGFR, CKD, urinary calculus, DBP, SBP, hypertension; Supplemental Table 8 and Figure 3). The directions of association of colocalizing traits were consistent with biological knowledge based on studies of uromodulin levels in urine, for example, higher serum uromodulin and higher risk of CKD (Figure 3). These observations are consistent with a common biological context of the *PRKAG2* and *UMOD*/*PDILT* loci in the pathophysiology of CKD, hypertension, and kidney stone disease, and with the earlier identification of the *PRKAG2* locus in GWAS of CKD (39). Conditional colocalization of 2 independent SNP sets at the *UMOD* locus further supported a shared genetic cause between the levels of circulating and urine uromodulin levels (Figure 3 and Supplemental Table 9).

There were several other examples of positive colocalizations supported by biological knowledge: first, genetic associations at the *B4GALNT2* locus with antibody-based circulating uromodulin colocalized with the odds of multiple gestation. This is consistent with a role of the B4GALNT2-mediated Sda antigen in embryo implantation in mice (40) (Figure 3). Second, SNPs at *SIGLEC9* associated with plasma aptamer uromodulin readout colocalized with levels of alkaline phosphatase in blood, which is in line with altered bone turnover described in a recent knockout mouse model of the homologous gene (41). These observations suggest that the aptamer-based assay is particularly well suited to generate insights into the generation of glycosylation residues that are present on uromodulin and how such glycosylation residues are recognized in the circulation. Tests of pairwise interactions of the 13 index SNPs, which could point toward nonadditive effects when the same pathway is affected, showed a significant interaction between genotype at the lead SNPs in *ST3GAL6* and *SIGLEC9* on plasma aptamer uromodulin readout (interaction *P* value = 3 × 10^–7^; Methods). These 2 genes are indeed functionally related, as *ST3GAL6* is involved in the synthesis of sialic acid moiety that is bound by SIGLEC9.

Additionally, we tested the aggregate effect of rare (MAF < 0.1%), potentially deleterious variants in the genes prioritized at each of the 13 uromodulin-associated genetic loci on 770 complex diseases (Methods). Using data from whole-exome sequencing of 173,688 UK Biobank participants, significant associations (*P* < 4.9 × 10^–6^) were identified between carrier status of rare *UMOD* variants and anemia of chronic disease (OR = 2.8, *P* = 6.1 × 10^–9^), hypertensive CKD (OR = 2.6, *P* = 1.8 × 10^–6^), and CKD (OR = 1.89, *P* = 2.0 × 10^–9^; Supplemental Table 10).

### Prioritization of causal genes in uromodulin-associated loci.

Colocalization analyses of the uromodulin association signals at all 13 significant loci were also performed with the expression of genes in *cis* based on transcriptome-wide RNA-sequencing of 36 non-brain tissues (GTEx Project V8) (31), tubulointerstitial and glomerular kidney tissue portions (42), as well as circulating plasma proteins (35) in order to prioritize the most likely causal genes (Methods). Plasma proteomics captures information about the abundance, structure, and context of circulating proteins and, when integrated with genomics, can reveal new insights into proteins that mediate genetic associations with complex traits and diseases (35, 43, 44). At least 1 positive colocalization with gene expression or plasma proteins was observed for most loci (Supplemental Table 11 and Supplemental Figure 7). For example, the association of genetic variants on chromosome 19 with uromodulin colocalized with their association with SIGLEC9 protein, further supporting that genetic variation in *SIGLEC9* relates to changes in the aptamer readout of circulating uromodulin. We confirmed that the aptamer readouts of plasma uromodulin and SIGLEC9 were correlated in the ARIC study (Spearman’s coefficient 0.53, *P* < 2.2 × 10^–16^).

Integration of colocalization evidence with additional sources of annotation (Methods) implicated *PRKAG2*, *B4GALNT2*, and *UMOD*/*PDILT* as the genes most likely causing the association with antibody-based uromodulin, and *CFH*, *MGAT5*, *ST3GAL6*, *HLA-DRB1*, *B4GALT1*, *ABO*, *DPP7*/*MAN1B1*, *ST3GAL4*, *ASGR1*/*ASGR2*, and *SIGLEC9* for the aptamer-based readout (Supplemental Table 12).

**Figure 4:**
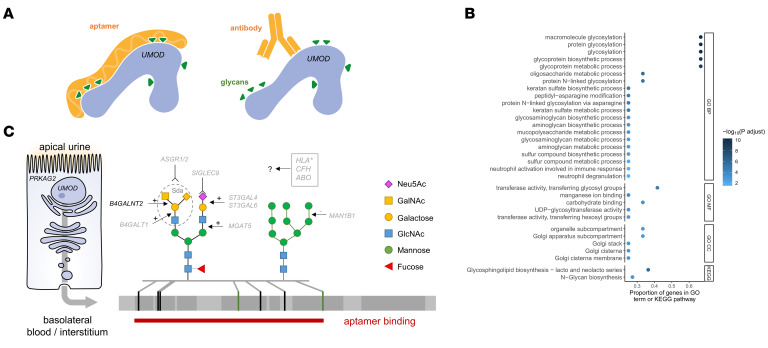
Biological context of genes associated with circulating uromodulin and conceptual model. (**A**) Schematic of antibody- and aptamer-based measurement of circulating uromodulin. (**B**) Dot plot shows Gene Ontology (GO) terms — grouped into 3 categories (BP, biological process; MF, molecular function; CC, cellular component) — and Kyoto Encyclopedia of Genes and Genomes (KEGG) pathways enriched for uromodulin-associated genes from the aptamer assay on the *y* axis. The *x* axis shows the proportion of the genes in the corresponding GO term or KEGG pathway. Only terms and pathways with more than 2 uromodulin-associated genes are displayed. The color intensity of the dots scales with the –log_10_(Benjamini-Hochberg–adjusted *P* value). (**C**) Conceptual model placing the most likely causal genes associated with circulating uromodulin into their biological context. Loci detected with the aptamer assay predominantly affect differential synthesis or recognition of glycan marks present on uromodulin. Gal, galactose; Glc, glucose; NAc, N-acetylgalactosamine; Neu5Ac, N-acetylneuraminic acid.

Antibody- and aptamer-based measurement of circulating uromodulin differ in their approach (Figure 4A). Whereas the antibody-based methods quantify abundance of the circulating protein as evidenced by the *cis* association at *UMOD/PDILT*, the aptamer-based assay may identify differences related to glycan marks known to be present on uromodulin and their receptors, for example, because such modifications may lead to differential aptamer binding. In order to detect any shared functions, processes, and pathways among the respective genes identified by each assay, enrichment analyses were performed (Methods). Terms and pathways related to protein glycosylation were highly enriched for genes identified via aptamer plasma uromodulin readout (Figure 4B), with the genes that drove the enrichment coding for enzymes and receptors involved in the biosynthesis and recognition of glycans, respectively (Figure 4C and Supplemental Table 13).

### B4GALNT p.Cys466Arg causes reduced enzyme function and processing.

**Figure 5:**
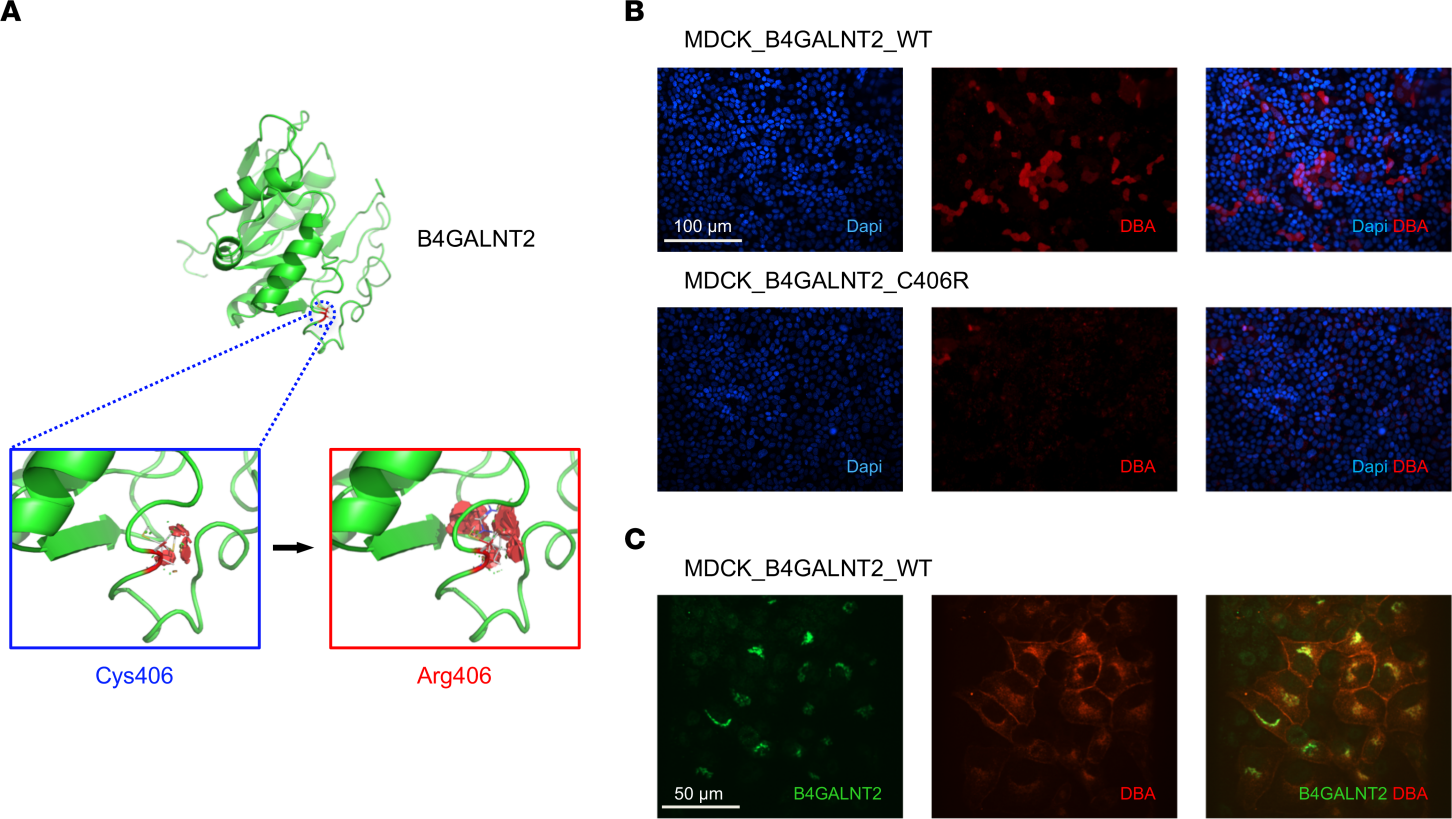
B4GALNT2 p.Cys466Arg is a functional allele. (**A**) Homology-based model of the tridimensional structure of B4GALNT2 enzyme. The partial sequence of B4GALNT2 isoform 2 (Uniprot Q8NHY0-2) containing the glycosyltransferase domain (residues 254–464) was analyzed with iTasser. The top-scoring model is shown. The position of cysteine 406 (corresponding to position 466 in B4GALNT2 isoform 1, Uniprot Q8NHY0-1) in the reference sequence is shown. The effect of the p.Cys406Arg substitution was analyzed in Pymol with the Mutagenesis Wizard function. For each isoform, the most likely stereoisomer is shown. Visible red disks indicate significant contacts and bumps. The arginine substitution at position 406 is predicted to increase steric clashes, destabilizing protein structure. (**B**) Representative immunofluorescence analysis showing DBA signal (red) on the plasma membrane of unpermeabilized MDCK cells, transiently transfected with WT or p.Cys406Arg human B4GALNT2 (*n* = 3). (**C**) Representative immunofluorescence analysis showing WT B4GALNT2 (green) and DBA (red) in stably transfected MDCK cells. DBA signal is mostly evident in B4GALNT2-expressing cells (*n* = 3).

To study the possible biological effect of rs7224888, we first generated a homology-based model of B4GALNT2. The arginine insertion at p.Cys466Arg was predicted to reduce protein structural stability by 3 programs (Pymol, Site Direct Mutation, Missense3D), likely as a consequence of the higher steric hindrance of arginine (Figure 5A and data not shown). To validate the in silico findings, we transfected Madin-Darby Canine Kidney (MDCK) cells with expression vectors for the 2 B4GALNT2 allelic variants. Two isoforms were reported for B4GALNT2, long and short (45). For this project, we used an already described expression vector for B4GALNT2 short isoform, where cysteine 466 corresponds to residue 406. A previous study (32) suggested that the p.Cys466Arg variant affects a region that glycosyltransferases typically use to interact with their substrate, impairing B4GALNT2 activity. Thus, we tested the activity of the enzyme isoforms (WT and Arg406) by taking advantage of the well-established interaction between the *Dolichos* *biflorus* agglutinin (DBA) and the Sda antigen (46). By using a rhodamine-labeled version of DBA, we observed a clear signal, mostly localized on the plasma membrane, in MDCK cells expressing WT B4GALNT2, while virtually no signal could be detected for Arg406-expressing cells (Figure 5B), confirming absent activity. Double staining of WT B4GALNT2 and DBA confirmed the specific presence of Sda antigen only in cells expressing the enzyme (Figure 5C).

**Figure 6:**
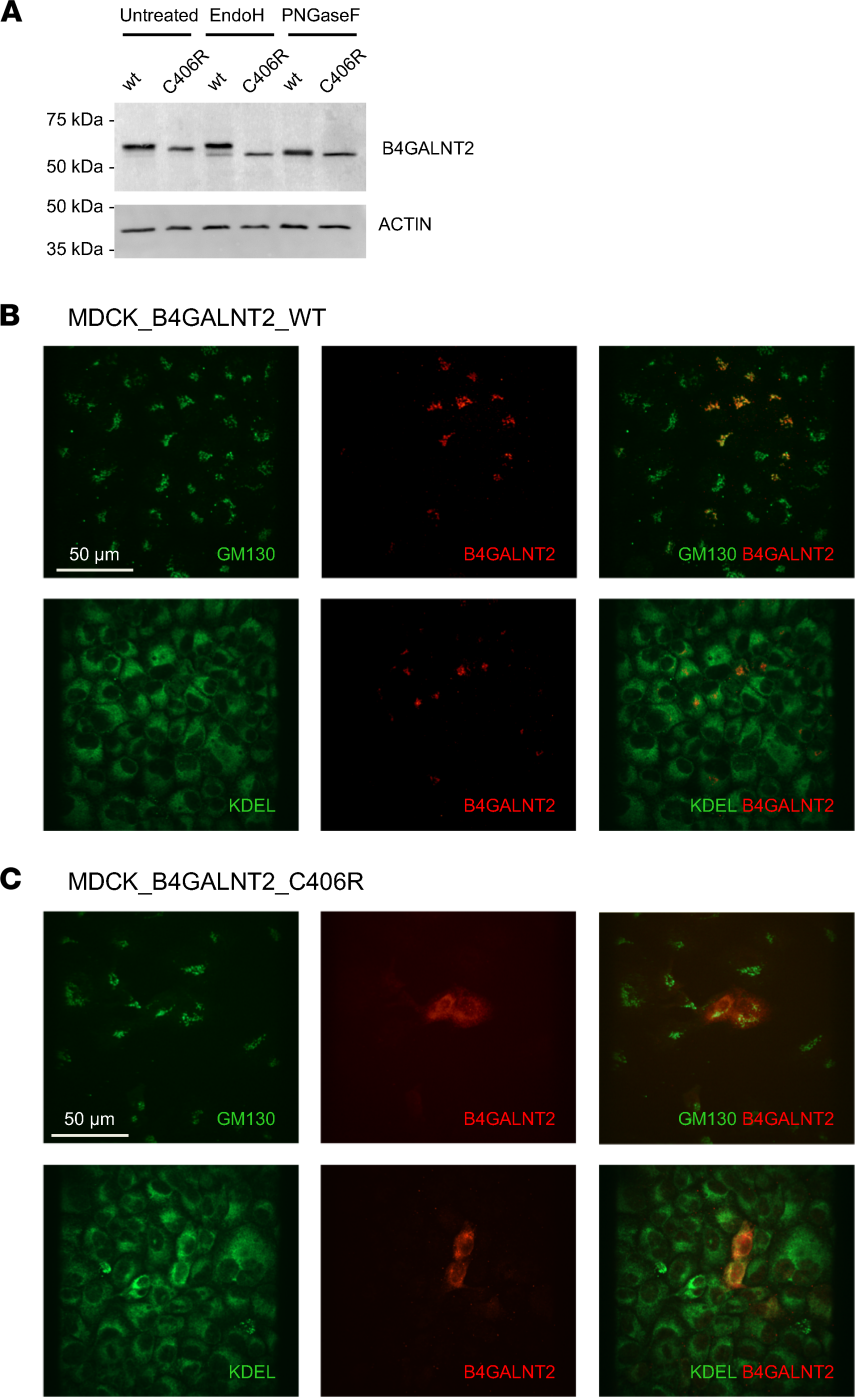
B4GALNT2 p.Cys466Arg is retained in the ER. (**A**) Representative Western blot analysis showing B4GALNT2 WT or p.Cys406Arg in stably transfected MDCK cell lysates, untreated or after deglycosylation with Endo H or PNGase F (*n* = 3). (**B** and **C**) Representative immunofluorescence analysis showing intracellular signal of WT (**B**) or p.Cys406Arg (**C**) B4GALNT2 (red) and GM130 (Golgi marker, green) or KDEL (ER marker, green) and merged pictures (*n* = 3).

Western blot analysis on cell lysates showed that Arg406 B4GALNT2 had a slightly reduced molecular weight compared with WT protein. Such difference is related to different glycosylation, due to retention of Arg406 B4GALNT2 in the endoplasmic reticulum (ER). Indeed, the lower band observed for the Arg406 isoform was fully sensitive to treatment with Endo H, a deglycosylating enzyme that is specific for high-mannose, ER-type N-glycans, while only a minor fraction of WT B4GALNT2 was cleaved by Endo H (Figure 6A). To substantiate this finding, we analyzed the intracellular localization of B4GALNT2 by immunofluorescence. While the WT isoform showed the expected predominant localization in the Golgi compartment, the Arg406 isoform fully colocalized with the ER marker KDEL, confirming its ER retention (Figure 6, B and C). These results demonstrate that the B4GALNT2 variant p.Cys466Arg is functional and leads to loss of B4GALNT2 function and ER retention, likely due to protein misfolding.

**Figure 7:**
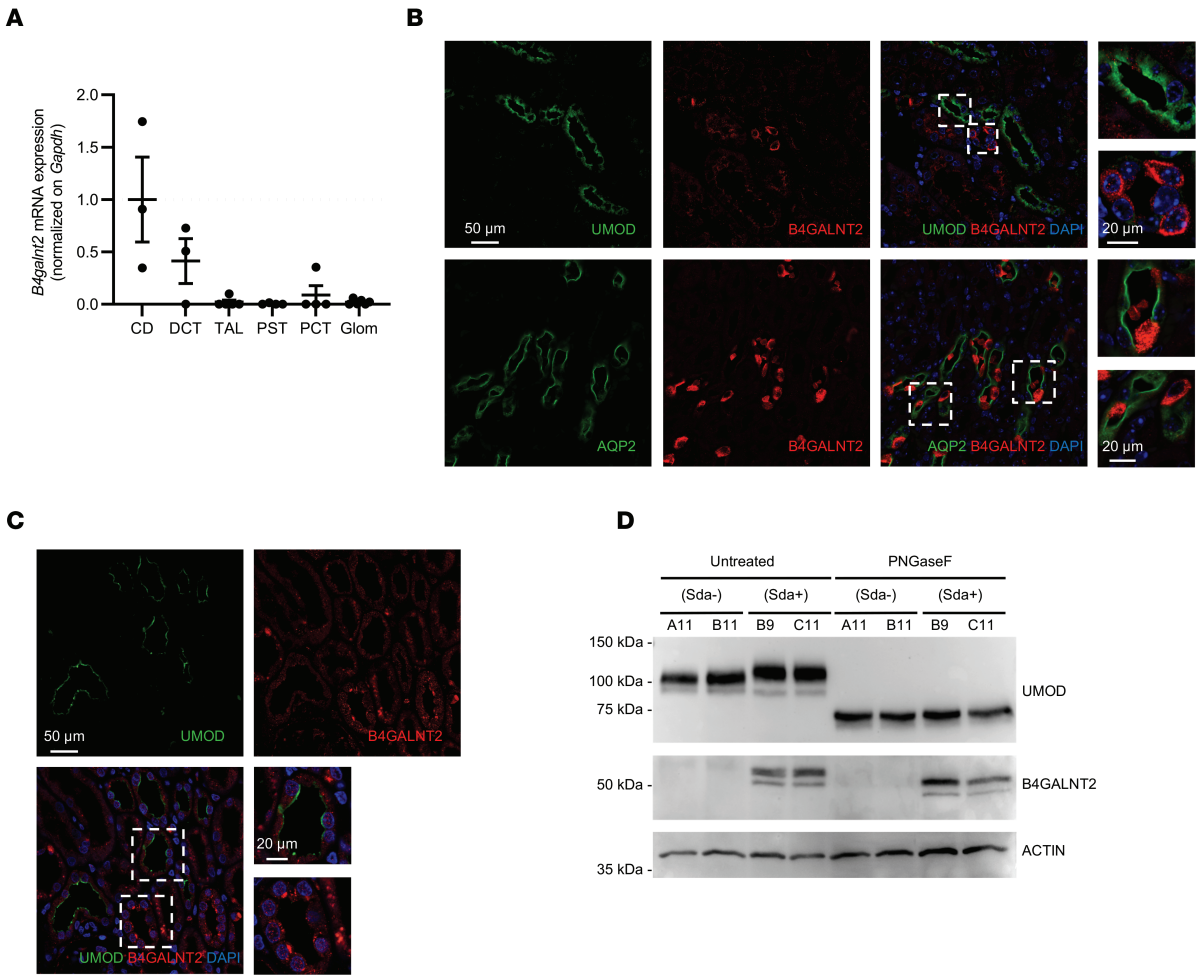
B4GALNT2 and uromodulin expression analysis. (**A**) RT-qPCR analysis of *B4galnt2* expression in isolated mouse nephron segments. Bars indicate mean ± SEM. *n* ≥ 3 fractions. Glom, glomerulus; PCT, proximal convoluted tubule; PST, proximal straight tubule; TAL, thick ascending limb; DCT, distal convoluted tubule; CD, collecting duct. (**B**) Upper panels: Immunofluorescence analysis showing UMOD (green) and B4GALNT2 (red) on paraffin-embedded kidney sections from WT mice (*n* = 2). Right panels show high-magnification pictures of UMOD-positive and UMOD-negative tubules. Nuclei are counterstained with DAPI. Lower panels: Immunofluorescence analysis showing AQP2 (green) and B4GALNT2 (red) on mouse kidney, showing a strong signal in the intercalated cells of collecting ducts. Right panels show high-magnification pictures of AQP2- and B4GALNT2-positive tubules. Nuclei are counterstained with DAPI. (**C**) Immunofluorescence analysis showing UMOD (green) and B4GALNT2 (red) on paraffin-embedded kidney sections from a normal human kidney. Right panels show high-magnification pictures of UMOD-positive and UMOD-negative tubules. Nuclei are counterstained with DAPI. (**D**) Western blot analysis showing uromodulin and B4GALNT2 in lysates of MDCK cell clones expressing UMOD with or without B4GALNT2 (see Methods), untreated or after deglycosylation with PNGase F. Actin is shown as a normalizer.

Previous in vitro studies demonstrated that an N-acetyl-β-d-galactosaminyltransferase activity present in microsomal preparations of guinea pig kidney transfers *N*-[^14^C]-acetylgalactosamine to N-linked glycans of UMOD for the synthesis of Sda antigen (47). To assess whether B4GALNT2 directly acts on uromodulin under physiological conditions, we first verified their coexpression in kidney cells using real-time reverse transcription quantitative PCR (RT-qPCR) on RNA extracted from micro-dissected mouse nephron segments and immunofluorescence on mouse and human kidney tissue (Methods). Real-time RT-qPCR demonstrated the presence of *B4GALNT2* transcript in TAL and DCT segments where uromodulin is expressed (Figure 7A). These data were confirmed by immunofluorescence analysis that showed a strong B4GALNT2 signal in collecting ducts (AQP2^+^) and a low but consistent signal in UMOD-positive cells (Figure 7B). B4GALNT2 expression in UMOD-positive cells was also confirmed in human kidney tissue (Figure 7C). To demonstrate the activity of B4GALNT2 on uromodulin glycosylation, we generated MDCK clones stably expressing uromodulin with/without B4GALNT2. Western blot analysis showed that uromodulin had a slightly increased molecular weight in lysates of B4GALNT2-positive cells that was due to different protein glycosylation, as demonstrated by removal of N-glycans through PNGase F treatment (Figure 7D).

**Figure 8:**
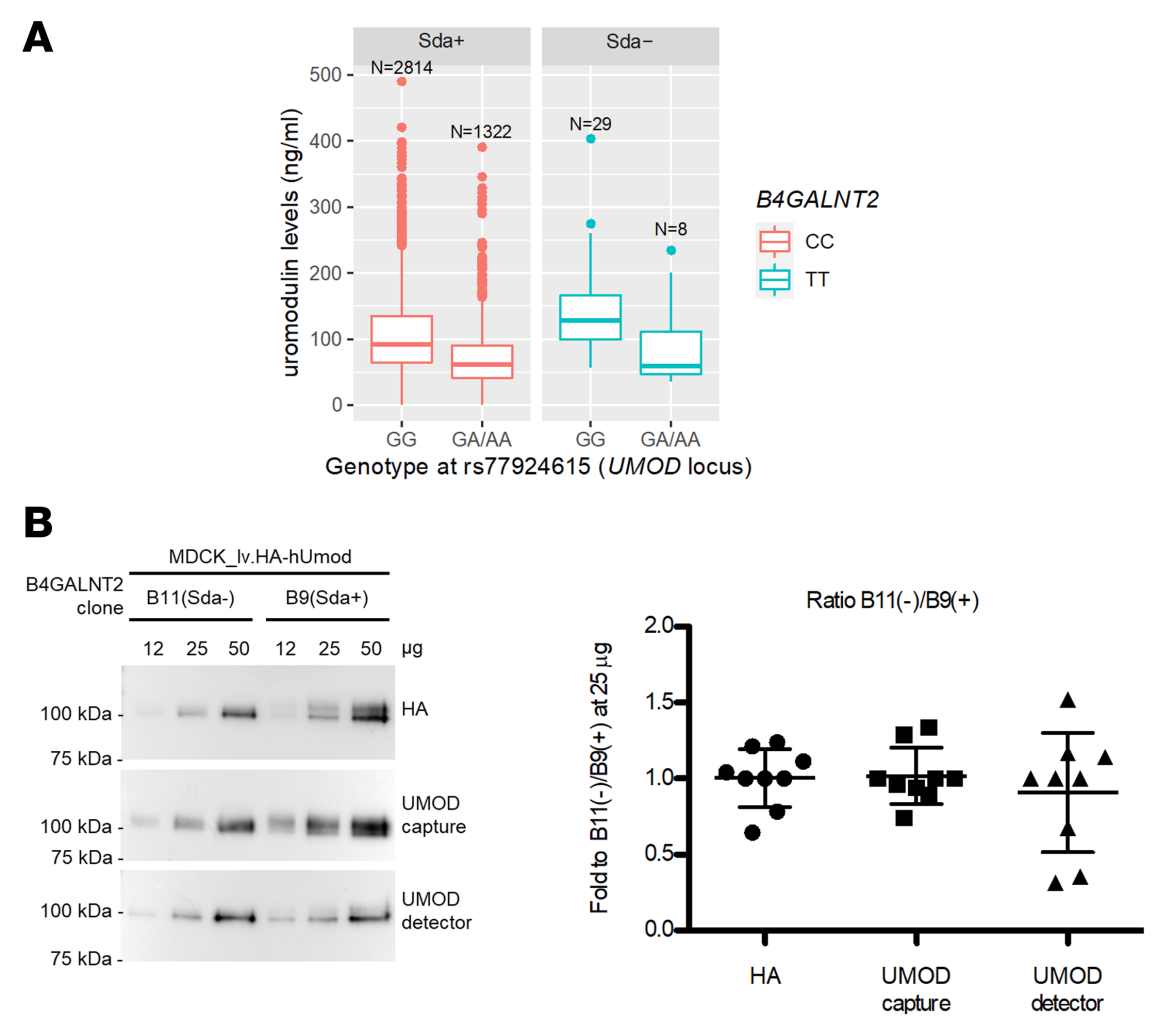
ELISA-based uromodulin quantification is not affected by presence of Sda antigen. (**A**) Uromodulin serum levels in individuals carrying GG or GA/AA genotype at *UMOD* variant rs77924615, stratified for their genotype at *B4GALNT2* variant rs7224888 (CC, Sda+; TT, Sda-). The expected differences in uromodulin levels are detected regardless of the presence/absence of Sda antigen. The start and end of boxes represent the 25th and 75th percentiles of the uromodulin distribution. The line inside the box represents the median, and the dots indicate outliers above the 75% + 1.5 × interquartile range of uromodulin values. (**B**) Representative Western blot analysis (left) and relative quantification (right) of uromodulin in lysates of MDCK cells transduced with lentiviral vector expressing HA-tagged uromodulin (lv.HA-hUMOD) and stably expressing B4GALNT2 (Sda+) or not (Sda-). The immunoreactivity of 3 different antibodies (anti-HA, and the 2 antibodies of the Euroimmun ELISA anti-UMOD capture and anti-UMOD detector) was assessed by loading and quantifying increasing amounts of cell lysate. Each value represents the ratio between B11 (negative clone) and B9 (positive clone) expressed as fold relative to the ratio measured with 25 μg of cell lysate (*n* = 3 independent experiments). Data are represented as vertical scatterplot expressed as mean ± SD (1-way ANOVA; *P* = 0.66). The ratios obtained for the different antibodies are comparable, suggesting similar immunoreactivity that is not modified by the presence of the Sda antigen.

Finally, we excluded that the association of B4GALNT2 loss of function with higher circulating uromodulin can be ascribed to altered immunoreactivity due to absence of the Sda antigen based on 2 observations. First, the quantitative, additive effect of *UMOD* variants on serum uromodulin was clearly detected regardless of the *B4GALNT2* genotype (Figure 8A). Second, the immunoreactivity of both ELISA antibodies did not differ from a reference antibody in detecting increasing amounts of uromodulin produced by cells expressing or not expressing B4GALNT2, hence carrying or not carrying the Sda antigen glycan moiety (Figure 8B).

## Discussion

This GWAS meta-analysis of circulating uromodulin using complementary antibody-based (*n* = 13,985) and aptamer-based (*n* = 18,070) assays has 4 principal findings. First, it identifies an upstream variant at the *UMOD*/*PDILT* locus for which integration with functional genomics data from primary human kidney tissue supports differential chromatin accessibility and transcription in cells synthesizing uromodulin as underlying its strong association with circulating and urine uromodulin, as well as CKD and hypertension. Second, shared association patterns of uromodulin-associated genes with complex traits and diseases are plentiful and place the *PRKAG2* and *UMOD* loci into the same context with respect to their associations with CKD, hypertension, and kidney stone disease. Third, the missense variant p.Cys466Arg in the uromodulin-glycosylating enzyme B4GALNT2 is a loss-of-function allele leading to higher levels of circulating uromodulin. Fourth, our study reveals enzymes that write glycan marks found on uromodulin and their receptors that may be related to uromodulin’s complex glycosylation pattern, function, and clearance.

Previous GWAS of circulating uromodulin quantified with antibody assays only identified the *UMOD* locus (23). At the *UMOD* locus, findings were consistent with those from urine (16) and a previous small GWAS of serum uromodulin (23); e.g., the major allele at the index SNP was associated with higher uromodulin levels. The generation of functional genomic data from kidney tissue now allowed for insights at this locus, by providing a plausible mechanism by which an intronic variant rs77924615 in the upstream gene *PDILT* is associated with uromodulin levels (16), despite the absence of *PDILT* transcription in kidney. Evidence for accessible chromatin at the SNP’s position solely in target kidney cell types for uromodulin synthesis, TAL and DCT cells, and mapping of rs77924615 and the functional *UMOD* promoter index SNP rs4293393 within the same topological associated domain, are indicative of a regulatory effect of this upstream variant on uromodulin transcription. This mechanism is also likely to underlie the reported associations of rs77924615 with urine uromodulin levels, kidney function, and CKD (7).

There are several potential explanations why a previous meta-analysis of urine uromodulin levels did not detect any of the other loci identified here despite a similar sample size (16). First, uromodulin occurs as a polymer in urine but is present as a monomer in blood. In addition, the 100-fold higher levels in urine as well as the high biological variability of urine concentration and composition may preclude the detection of slight variations that can be observed in plasma. Our study supports a shared genetic basis for urine and circulating uromodulin levels, but the index variant rs77924615 explained more than 10 times as much of the uromodulin variance in the circulation compared with the urine. Thus, circulating uromodulin may be a more attractive biomarker to estimate uromodulin production in the kidney. Second, it is conceivable that receptors recognizing glycan marks present on uromodulin, potentially affecting its stability or clearance, differ between urine and the circulation. Third, previous urine studies did not use aptamer-based assays. Our results suggest that the aptamer detects genetic loci related to the writing of glycans that are present on many secreted glycoproteins, including uromodulin, and their recognition in the circulation, rather than representing the abundance of the intact protein in blood. Future validation of the uromodulin aptamer, and investigations of how the amount of uromodulin protein relates to its glycosylation patterns, are of interest. Regardless, the aptamer readout carries complementary information by delivering insights into the glycan component of this important glycoprotein. Despite the distinct set of loci detected with the 2 assays, there are also connections: the *ABO*-encoded glycosyltransferase acts on precursor chains that are also substrates of the enzymes encoded by *ST3GAL4* and *ST3GAL6* to synthesize type 2 monosialyl-galactosylgloboside that is used for the synthesis of sialyl Lewis X antigen, and by B4GALNT2 for the synthesis of the Sda antigen (48, 49).

Concerning B4GALNT2, our functional studies demonstrate that the p.Cys466Arg variant leads to retention of the Arg466 isoform in the ER and its absence in the Golgi compartment. This is likely due to protein misfolding, as suggested by prediction analysis of the effect of the missense change on B4GALNT2 structural stability. The consequence, loss of protein function, is demonstrated by virtual absence of DBA positivity. These data are consistent with and provide a mechanistic explanation for previous results showing that the Arg466 variant is statistically correlated with absence of the Sda antigen (32).

The role of B4GALNT2 in uromodulin glycosylation is supported by the proteins’ demonstrated coexpression in TAL and DCT segments in mouse and human kidney. Moreover, we show that expression of B4GALNT2 in cells expressing uromodulin leads to addition of a glycan moiety, presumably the Sda antigen. The functional role of the Sda antigen on uromodulin is not known. Through the addition of β1,4-linked GalNAc, it may hinder binding of bacterial adhesins and, hence, have a role in pathogen resistance. There are several potential explanations for how B4GALNT2 loss of function, i.e., absence of the Sda antigen, may lead to higher serum levels of uromodulin as quantified by antibody. Neither our experimental nor our population study data provide evidence for an altered immunoreactivity due to absence of the Sda antigen, making this an unlikely option. It is conceivable that the presence of the Sda antigen is associated with lower stability of circulating uromodulin, as observed for von Willebrand factor through a mechanism that depends on asialoglycoprotein receptor (ASGPR) activity (50). Alternatively, the absence of the Sda antigen may influence uromodulin polarized trafficking, partly redirecting the protein toward the basolateral membrane and from there to the circulation.

Common variants at the *PRKAG2* locus showed a striking similarity to those at *UMOD* with respect to shared disease association patterns. Considering that we tested for colocalization with hundreds of human traits and diseases, these mirroring patterns are extremely unlikely to result from chance. Our results imply that genetic variants at *PRKAG2* are associated with higher risk for CKD and hypertension, as well as lower risk for kidney stone disease, through the same biological context as *UMOD*. *PRKAG2* codes for the regulatory γ subunit of the AMP-activated protein kinase (AMPK), an enzyme with a key role in regulating multiple processes related to cellular energy metabolism. AMPK has been described to phosphorylate the kidney-specific Na^+^-K^+^-2Cl^–^ cotransporter (51), the molecular link between CKD-associated *UMOD* variants and hypertension (15). However, although this connection is biologically plausible, we cannot exclude that *GALNT11*, *GALNT5*, or other genes or elements in the *PRKAG2* locus represent the causal link.

The loci identified through aptamer readout point toward the importance of uromodulin glycosylation in general and sialylation in particular. SIGLEC9 receptor binding to uromodulin depends on the presence of terminal sialic acid on uromodulin N-glycans (37). ASGPR, mainly expressed in liver hepatocytes and to a lesser extent in several cell types, including monocytes (52), mediates binding, endocytosis, and degradation of glycoproteins with decreased sialylation and exposed terminal GalNAc residues (52–55). An observed 4-fold increase of circulating uromodulin in mice upon ablation of Asgr2 and the mannose receptor (56) as well as the association between a rare *ASGR2* variant and the aptamer uromodulin readout in our study raise the possibility that circulating uromodulin may be another glycoprotein the ASGPR recognizes. The function of SIGLEC9 and ASGPR intersects with that of the ST3Gal family of sialyltransferases, which add sialic acid in alpha2,3 linkage to terminal galactose, thereby generating potential SIGLEC9 ligands, while masking ASGPR ligands (53, 57–59). Overall, these data point at a clear functional relationship between SIGLEC9 and ASGPR, potentially involved in uromodulin-mediated immunomodulatory signaling and clearance, and ST3GAL4 and 6, modulating such activities through sialylation of glycan moieties.

Our study also provides insights of potential clinical relevance. It indicates that a common genetic basis of urine uromodulin levels with higher risk of CKD and hypertension extends to circulating uromodulin levels and identifies kidney cell type–specific regulation of uromodulin expression as a mechanism. Interventions aimed at reducing uromodulin synthesis can therefore be expected to have concomitant effects on both urine and serum levels of the protein, which may be of importance given its emerging systemic relevance. The association of genetic variants at *PRKAG2* with higher risk for CKD and hypertension as well as lower risk for kidney stone disease suggests a biological link between PRKAG2 and UMOD and suggest that PRKAG2 represents another target to modulate uromodulin-mediated risk of CKD. Finally, therapeutic targeting of (a)sialoprotein receptors may affect circulating uromodulin, possibly modulating crosstalk between the kidney and the innate and adaptive immune system.

In conclusion, our study provides human genetic evidence of pathway members of uromodulin and delivers insights into its determinants and systemic role in the circulation.

## Methods

### Study design and participants.

Seven prospective studies participated in the genome-wide analyses of serum/plasma uromodulin levels (Supplemental Table 1): the CHS (60), the GCKD study (61), the KORA study (62), the LURIC study (63), the ORIGIN Trial (64), the ARIC study (65), and the Fenland study (66). Each study contributed data from EA participants. Data from AA and HIS participants were contributed by the CHS study (AA) and the ORIGIN Trial (AA, HIS). The ARIC, CHS, Fenland, and KORA studies have a population-based design, the GCKD study recruited patients with CKD, the LURIC study recruited patients with cardiovascular disease, and the ORIGIN Trial recruited patients with impaired glucose tolerance or early type 2 diabetes. Demographic information including age and sex was collected using standardized procedures. The eGFR was calculated from isotope dilution mass spectrometry–traceable serum creatinine measurements using the 4-variable CKDEpi equation (7).

### Genotyping and imputation.

Details about genotyping and imputation in each of the 7 studies are provided in Supplemental Table 2. In brief, all samples were genotyped for genome-wide SNPs using Illumina or Affymetrix arrays and called using commercial software. Variant-level quality control and cleaning included removal for low call rate and deviation from Hardy-Weinberg equilibrium. Genotype imputation was then performed using phasing and imputation software, based on the Trans-Omics for Precision Medicine haplotypes version r2 (ARIC) or the Haplotype Reference Consortium (HRC) haplotypes version r1.1 (all other studies) reference panels.

### Uromodulin quantification.

The CHS, GCKD, KORA, and LURIC studies quantified uromodulin from serum using a commercial ELISA (23, 30, 67, 68) (Euroimmun, Medizinische Labordiagnostika AG). The assay is based on a colorimetric sandwich immunoassay, in which the capture antibody was a mouse monoclonal antibody against human uromodulin, and the detection antibody was a biotinylated mouse monoclonal antibody against human uromodulin. The intra-assay precision of the assay at 30–214 ng/mL was 1.8%–3.2%, and the interassay precision at 35–228 ng/mL 6.6% to 7.8% (21). The ORIGIN Trial measured serum uromodulin using an immunoassay that is part of the Human DiscoveryMAP panel (Myriad RBM Inc.) with a biotinylated polyclonal detection antibody against human uromodulin (69). In the ARIC and Fenland studies, uromodulin was quantified as part of plasma proteome profiling using the SomaScan assay (Seq-ID 9451-20), a multiplexed modified DNA-based aptamer technology by SomaLogic as described previously (43). The aptamer was raised against amino acids 24–611 of human uromodulin (NP_001008390.1). Its signal-to-noise ratio was 114, and its intra- and interassay coefficients of variation were 3.2% and 3.6%, respectively.

### GWAS of serum uromodulin levels and meta-analyses.

Each study performed 4 sets of GWAS according to a prespecified analysis plan: a primary analysis, in which rank-based inverse normal transformed age-, sex-, and eGFR-adjusted residuals of uromodulin levels were used as the dependent variable and regressed on genotypes as the predictor, controlling for principal components. In addition, 3 secondary analyses were carried out: the primary analysis was repeated separately for men and women (without adjustment for sex and only when at least 50 participants were available), and a sex-combined analysis was performed in which the residuals were not adjusted for eGFR. Studies with participants of different ancestries performed separate GWAS for each ancestry group. Sex-stratified GWAS of chromosome X markers, assuming X inactivation and an additive model, were only available from the CHS and GCKD studies. Genome-wide summary statistics were collected in a prespecified format and uploaded to a central server for meta-analysis.

Prior to meta-analysis, GWAS summary statistics from individual studies were subjected to thorough quality control using GWAtoolbox (70). The variant identifiers in each GWAS file were harmonized to the format chromosome:position:ref:alt, where ref and alt are the REF and ALT alleles in the HRC r1.1 reference site file. Genome-wide summary statistics were combined for studies with antibody-based uromodulin quantification using inverse-variance-weighted meta-analysis of effect estimates. Previous meta-analyses of uromodulin levels in urine quantified from ELISA and the RBM immunoassay showed little heterogeneity and comparable results when using inverse variance-weighted and sample size–weighted meta-analysis (16). The primary meta-analysis was a trans-ethnic analysis, combining data from EA, AA, and HIS participants, and performed using metal (71). Sex-specific summary statistics of chromosome X were combined via meta-analysis. Genomic control was applied to individual GWAS files when the inflation parameter was more than 1. Genome-wide significance was defined as 2-sided *P* < 5 × 10^–8^. EA-specific meta-analyses were also performed, as EA participants were the largest subsample. SNP-based heritability was estimated using LDSC v1.0.1 with the option --h2. Precomputed LD scores from 1000 Genomes European data were used as reference. Input files for LDSC were GWAS summary statistics from the primary association analyses filtered for MAF > 0.01 (antibody-based assay) or MAF > 0.005 (aptamer-based assay).

### Downstream characterization of GWAS meta-analysis results.

Several complementary approaches to characterize genetic loci identified through genome-wide screens of circulating uromodulin were employed, with detailed methods described in Supplemental Methods. These include (i) associations with urine uromodulin levels; (ii) annotation, enrichment analyses, and functional genomics; (iii) independent SNP selection, statistical fine mapping, and credible set annotation; (iv) colocalization with gene expression, plasma protein levels, biomarkers, and diseases; (v) phenome-wide association studies of serum uromodulin levels; as well as (vi) gene-by-gene interaction analyses.

### Three-dimensional modeling of B4GALNT2 and prediction of the effect of p.Cys466Arg.

The sequence of B4GALNT2 isoform 2 (Uniprot Q8NHY0-2) was analyzed in PFAM (http://pfam.xfam.org) to map functional domains. The region 254–464 containing the glycosyltransferase domain was analyzed in iTasser (72) to generate a homology-based 3D model. The effect of the p.Cys466Arg substitution was analyzed in Pymol (version 2.3.4) (Schrödinger) with the Mutagenesis Wizard function, in Site Direct Mutator (73), and in Missense3D (74).

### Experimental studies of B4GALNT2.

Detailed information regarding functional studies of B4GALNT2 (constructs, cell line and culture conditions, protein extracts and Western blot analysis, RNA isolation and RT-qPCR, immunofluorescence analysis, antibodies) are reported in Supplemental Methods. Protein and RNA extracts, Western blot, RT-qPCR, and immunofluorescence analyses were carried out as previously described (9, 75).

### Data availability.

Genome-wide summary statistics of the meta-analyses of circulating uromodulin are available at web page https://nxc-1453.imbi.uni-freiburg.de/s/gReQNkMJtkYYLxa Publicly available single-nucleus ATAC-seq data were downloaded from https://susztaklab.com/human_kidney/igv/ and generated as described previously (76). 

### Statistics.

Genome-wide significance was defined as 2-sided *P* < 5 × 10^–8^. ANOVA was used for the statistical analysis of some of the experimental data, as specified in figure legends.

### Study approval.

All participants of CHS, GCKD, KORA, LURIC, ORIGIN, ARIC, and Fenland studies provided written informed consent, and the studies were approved by their local ethics committees as outlined in their respective design publications (60–66). The use of human kidney biopsies in experimental studies of B4GALNT2 has been approved by the UCLouvain Ethical Review Board (Brussels, Belgium). Human kidney tissues used in functional genomics were collected in deidentified fashion through Northwest Biotrust at the University of Washington Medical Center (Seattle, Washington, USA) with local IRB approval (Study 1297).

All mouse experiments were performed in accordance with the ethical guidelines at University of Zurich (Zurich, Switzerland) and the legislation of animal care and experimentation of Canton Zurich (Kanton Zürich Gesundheitsdirektion Veterinäramt; protocol ZH049/17).

## Author contributions

YL, YC, FC, LR, and AK conceived and designed the study; YL, YC, MRC, M Pietzner, NQHN, NS, MLB, MEK, BG, P Sekula, IS, P Schlosser, MW, SA, LR, and AK performed statistical analysis; MEK, M Pigeyre, JAB, MEG, CH, UTS, BKK, FK, AP, JS, DS, CT, KUE, CG, EB, BMP, JC, CL, BY, and AK managed an individual study; JS, DS, CT, WM, GP, and JES measured uromodulin; YL, YC, MEK, NS, SH, CBJ, and MW integrated bioinformatic data; YL, YC, FC, GS, PJO, SA, OD, LR, and AK interpreted results; FC, GS, SA, OD, and LR performed experimental studies; YL, YC, FC, GS, SA, LR, and AK wrote the initial manuscript draft; and all authors critically reviewed the manuscript. The order of the co–first authors was determined based on the overall analytical and conceptual contribution to the project.

## Supplementary Material

Supplemental data

Supplemental tables 1-13

## Figures and Tables

**Table 1:**
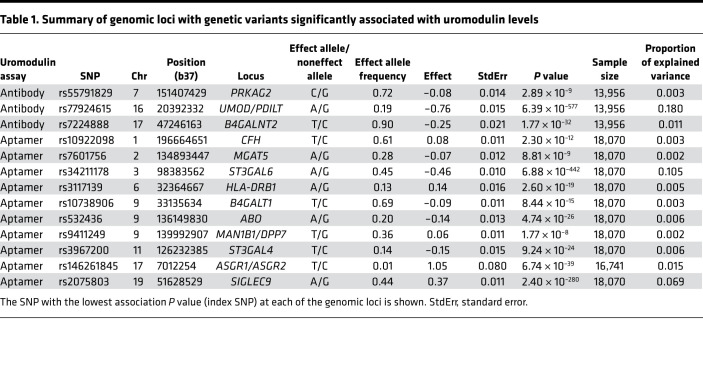
Summary of genomic loci with genetic variants significantly associated with uromodulin levels

## Version 1 04/21/2022

In-Press Preview

## Version 2 05/23/2022

Electronic publication
