# Supplementary material for: Genome-wide studies reveal factors associated with circulating uromodulin and its relationships to complex diseases
Source: JCI Insight. 2022 May 23;7(10):e157035. doi: 10.1172/jci.insight.157035 (PMC9220927; doi:10.1172/jci.insight.157035)
Supplement: Supplemental data [file jciinsight-7-157035-s128.pdf]

Genome-wide studies reveal factors associated with circulating uromodulin and its relations with complex diseases

Yong Li, Yurong Cheng, Francesco Consolato, Guglielmo Schiano, Michael R. Chong, Maik Pietzner, Ngoc Quynh H. Nguyen, Nora Scherer, Mary L. Biggs, Marcus E. Kleber, Stefan Haug, Burulça Göçmen, Marie Pigeyre, Peggy Sekula, Inga Steinbrenner, Pascal Schlosser, Christina B. Joseph, Jennifer A. Brody, Morgan E. Grams, Caroline Hayward, Ulla T. Schultheiss, Bernhard K. Krämer, Florian Kronenberg, Annette Peters, Jochen Seissler, Dominik Steubl, Cornelia Then, Matthias Wuttke, Winfried März, Kai-Uwe Eckardt, Christian Gieger, Eric Boerwinkle, Bruce M. Psaty, Josef Coresh, Peter J. Oefner, Guillaume Pare, Claudia Langenberg, Jürgen E. Scherberich, Bing Yu, Shreeram Akilesh, Olivier Devuyst, Luca Rampoldi, Anna Köttgen

Supplementary Material

Table of Contents

Supplementary Methods 2
Downstream characterization of GWAS meta-analysis findings 2
Association with urine uromodulin levels 2
Annotation, Enrichment Analyses and Functional Genomics 2
Independent SNP selection, statistical fine mapping and credible set annotation 4
Colocalization with gene expression, plasma protein levels, biomarkers and diseases ... 5
PheWAS of serum uromodulin levels 7
Gene-by-gene interaction analyses 8
Experimental studies of B4GALNT2 9
Supplementary Figures 13
Supplementary References 24

Supplementary Methods

Downstream characterization of GWAS meta-analysis findings

Association with urine uromodulin levels

The association between the index SNP at each genome-wide significant locus and the concentrations of uromodulin in the urine were queried in a GWAS meta-analysis of up to 29,512 participants of 13 cohorts (1). Urine uromodulin was quantified with a previously described ELISA (2) in all but one study, that used the same RBM immunoassay as used by the ORIGIN study in this project. Summary statistics for the index SNPs were extracted from results of an inverse-variance weighted fixed-effects meta-analysis of GWAS of urinary uromodulin levels based on well imputed and quality-controlled genotypes (1).

Annotation, enrichment analyses and functional genomics

SNPs were annotated by querying the SNiPA database (3) version 3.4 (November 2020). In SNiPA v3.4, the variant effect annotations were based on the Ensembl 100 release. Based on SNiPA annotations and colocalization analyses, we performed gene prioritization at each locus by assigning each potential candidate gene evidence scores. The prioritization procedure has been described previously (4), with additional evidence scores for colocalization with eQTL and pQTLs that were prioritized over other sources of evidence. In the case of ties, the closest gene was assigned. As gene prioritization from GWAS signals is not always unambiguous, for example because of incomplete tissue representation in some of the underlying data sources, we report two most likely causal genes at three of the loci to account for uncertainty. Gene Ontology and KEGG pathway enrichment analyses based on the resulting 16 potentially causal genes were performed separately for the 4 genes from the

antibody and the 12 genes from the aptamer-based assay using the R package clusterProfiler (5) with default parameters. Statistically significant enrichment was defined as Benjamini-Hochberg-adjusted p-value <0.05. Results using the 13 entirely automatically assigned most plausible gene at each locus or the 13 biologically most plausible genes were essentially the same (data not shown).

Macroscopically identifiable cortex and medulla were obtained from grossly uninvolved tumor-adjacent normal tissue in nephrectomy specimens. RNA extraction and RNA-seq was performed by GeneWiz. The paired end fastq files were trimmed and aligned to human reference genome sequence hg38 using STAR 2.7.5b (6) with parameters --outFilterIntronMotifs RemoveNoncanonical --outFilterMismatchNoverReadLmax 0.04 using annotation file Homo_sapiens.GRCh38.100.gtf downloaded from Ensembl database. The number of reads aligned to each exon (feature) was counted using *featureCounts* from subread package (7). Read normalized density tracks (.bw) were visualized in the UCSC Genome Browser.

ATAC-seq was performed on snap-frozen samples of human kidney cortex by ActiveMotif and aligned to hg38 reference genome using BWA with default settings. Read depth was normalized by random downsampling to the level of the sample with lowest coverage. Peaks were called using MACS 2.1.0 at a cutoff of p-value 1e-7, without control file, and with the -nomodel option. Peak filtering was performed by removing false ChIP-Seq peaks as defined within the ENCODE blacklist.

Hi-C was performed on macro-dissected kidney cortex from a 74 year-old man. Approximately 0.5 cm^3 of tissue was minced with a razor blade and fixed and nutated for 20 minutes in 10 mls of a 1:10 dilution of 10% neutral buffered formalin (Fisher). Following this, the formalin was quenched by direct addition of 0.1 g of glycine (Sigma) and nutation for

another 15 minutes. The tissue fragments were submitted to Phase Genomics for Hi-C library preparation using their Human Hi-C Kit. A total of 369,485,049 2x150 bp read pairs were sequenced from the resulting library on an Illumina HiSeq 4000. Topology associated domain (TAD) calling was performed using the DomainCaller algorithm at 50kbp resolution with default parameters. Chromosomal contacts (binned to 10kb resolution) were generated using the Phase Genomics Matlock tool (https://github.com/phasegenomics/matlock). Contact matrices (.hic files) were visualized using the Juicebox application (https://www.aidenlab.org/juicebox/).

Publicly available single-nucleus ATAC-seq data generated from 12,720 human kidney cells (8) were obtained at https://susztaklab.com/human_kidney/igv/. Open chromatin peaks in different kidney cell types were saved and displayed together with the bulk RNA-seq and ATAC-seq tracks based on the corresponding data we generated from human kidney cortex and medulla.

Independent SNP selection, statistical fine mapping and credible set annotation

In order to identify additional independent SNPs other than the index SNP at each associated locus, conditional analysis using GCTA-COJO (9) were performed as described previously (10) using European ancestry GWAS summary statistics as input. HRC-imputed genotypes of the GCKD study were used as the LD reference. This reference panel contained genotypes of 23,215,029 variants with imputation quality >0.3 from 5,034 individuals.

Genomic regions for fine-mapping were defined as 1MB windows around each genome-wide significant index SNP. Overlapping regions were identified and merged at chromosome 3, 6 and 16. Within each region, a stepwise forward selection algorithm implemented in GCTA COJO-Slct was applied to the summary statistics, with collinearity value

of 0.1 to be more conservative than the default. Additional, independently genome-wide significant SNPs were defined as those with p<5e-08 after conditioning on genotypes at all previously identified SNPs in the same region. Subsequently, conditional association statistics were generated for regions with multiple independent SNPs using GCTA COJO-Cond (10). The R package gtx (https://github.com/tobyjohnson/gtx) implementing Wakefield's formula was used to derive approximate Bayes factors. Based on the effect size distribution in the uromodulin meta-analysis, we chose 0.034 as the standard deviation prior. The posterior inclusion probability (PIP) value for each SNP in the evaluated region was calculated, ranked, and 99% credible sets were constructed by adding SNPs until their cumulative PIP reached 0.99.

Colocalization with gene expression, plasma protein levels, biomarkers and diseases

To gain insights into the molecular basis of the identified genetic associations and to determine the most likely causal gene at each locus, we performed colocalization analyses of uromodulin-associated SNPs from the European ancestry participants with their respective associations from GWAS summary statistics of gene expression, plasma protein levels, biomarkers and diseases. The precomputed eQTL data from the GTEx Project V8 (11) (36 non-brain tissues) and the NEPTUNE study (12) (NephQTL from tubulo-interstitial and glomerular kidney portions) were downloaded from the respective web sites. GTEx brain tissues were excluded from the analysis. Association summary statistics of plasma protein levels (2,751 proteins represented by 3,022 SOMAmers) (*cis*-pQTL) were downloaded from http://www.phpc.cam.ac.uk/ceu/proteins/. Colocalization analysis was also performed between the uromodulin-associated loci identified with the antibody-based assay with the levels of any proteins with which the index SNP showed association in *trans*, which was true

for the QSOX2 protein (*trans*-pQTL). Phenotypes of 30 biomarkers were from UK Biobank (application #20272) and GWAS were performed using BOLT-LMM. Precomputed associations with 1,404 diseases and binary traits as defined by PheCodes from the UK Biobank were downloaded from https://www.leelabsg.org/resources. Additionally, we performed colocalization with five *UMOD*-related traits, namely eGFR, CKD, SBP, DBP and urine uromodulin obtained from the CKDGen Consortium (10, 13), ICBP consortium (14), and from Joseph *et al*. (1).

The detailed procedure of colocalization with eQTL and pQTL has been described previously (4). A *cis* window was defined by extending the corresponding gene region by 500 kb upstream and downstream. When there was at least one *cis*-eQTL or *cis*-pQTL with P<0.001 within ±100 kb of a uromodulin-associated index SNP, colocalization analyses were performed within the eQTL/pQTL *cis* window as defined in their primary publications (11, 12, 15). For colocalization with biomarkers and diseases, a colocalization analysis was performed within ±500 kb of an index SNP, when at least one variant of MAF >0.01 had a p-value<0.001. The 'coloc.fast' function from the R package gtx with default parameters and prior definitions were used, an adaptation of the colocalization method introduced by Giambartolomei (16). Positive colocalization was defined when the posterior probability of a single shared variant underlying the association signals for both traits (H4) was >0.8 (4).

Colocalization using marginal association statistics did not yield positive colocalization with urine uromodulin. Given that the association patterns at the *UMOD/PDILT* locus are however very similar, and two independent association signals with serum uromodulin were detected at this locus, conditional colocalization analysis with urine uromodulin levels were performed (17). After selecting independent SNPs associated with urine uromodulin as described in "Independent SNP selection", conditional association statistics were generated

separately for serum and urine uromodulin for each independent selected SNP by conditioning on the other selected independent SNPs. Colocalization analyses were subsequently performed for each pair of independent SNPs in serum and urine uromodulin using the conditional association statistics as input.

PheWAS of serum uromodulin levels

Individual-level UK Biobank data was accessed as part of application #15255. Whole-exome sequencing (WES) derived genotypes (UKB data field: 23155) were obtained for 200,643 UK Biobank participants using the gfetch utility. Quality control of WES data included the removal of (i) samples who withdrew consent, (ii) monomorphic variants, (iii) variants with missingness exceeding 10%, (iv) samples with missingness exceeding 1%, (v) samples exhibiting discrepancies between genetic and self-reported sex or ethnicity, (vi) variants deviating from Hardy-Weinberg equilibrium, (vii) samples of non-Caucasian ancestry (self-reported British, Irish, or "Other White" individuals who clustered with Europeans in PCA analyses), and (viii) samples exhibiting third degree or closer relatedness, and (ix) non-coding variants, resulting in 173,688 samples. Further variant selection was performed to retain variants that were rare (MAF <0.001), non-synonymous, and predicted to be clinically deleterious by Mendelian Clinically Applicable Pathogenicity (M-CAP) v.1.4 scores (or were highly disruptive variant types including frameshift indel, stopgain, stoploss, or splicing). Rare variant phenome-wide association testing was performed for prioritized genes in the eight GWAS loci (*PRKAG2*, *GALNTL5*, *UMOD*, *PDILT*, *B4GALNT2*, *ST3GAL6*, *C2*, *HLA-DQB1*, *HLA-DRB1*, *HLA-DRB5*, *ST3GAL4*, and *SIGLEC9*). *ABO* was not tested due to a low total minor allele count (<10). For each gene, rare allele counts were added per sample constituting the rare allele burden. Association testing between rare allele burden (independent variable) and disease

status (dependent variable) was performed using logistic regression in the statistical programming language, "R". Model covariates included age, age^2, sex, and 40 genetic principal components. Disease status was defined using the "PheCode" classification scheme to aggregate ICD-10 codes from hospital episodes (field ID 41270), death registry (field ID 40001 and 40002), and cancer registry (field ID 40006) records. PheCodes with a minimum case sample size of 300 were considered, leading to 770 PheCodes tested per gene. Statistical significance was defined as p<5.0e-06 [0.05/(770*13 loci)].

Gene-by-gene interaction analyses

To detect potential genetic interactions, pairwise interaction analyses between the 13 index SNPs were performed in three datasets: first, genetic interactions on antibody-based serum uromodulin levels were investigated in the GCKD study. Second, genetic interactions on aptamer-based serum uromodulin levels were investigated in the ARIC and Fenland study and combined via fixed-effects inverse variance weighted meta-analysis. Third, genetic interactions on seven phenotype domains (CKD [ICD-10 N18], creatinine [30700-0.0], cystatin C [30720-0.0]; hypertension [I10], mean of systolic [4080-0.0/1] and diastolic [4079-0.0/1] blood pressure; gout [M10.0, M10.9]; purpura [D69] and other coagulation defects [D68]; urinary tract infections [N39.0]; alkaline phosphatase levels [30610-0.0]; and calculus of kidney and ureter [N20]) among up to 337,126 unrelated UK Biobank participants of white British ancestry with available genetic principal components and without excess heterozygosity or gonosomal aneuploidy (18), using application #64806. Linear regression models were fitted using R v4.0.5, regressing each pair of SNPs on age, sex, and eGFR-adjusted residuals of serum uromodulin (GCKD, ARIC, Fenland) or age, sex, genotyping array and the first 20 principal components (UKB) to obtain main effects per SNP and their interaction

effect. Statistically significant interaction was defined as interaction p-value <9.2e-05 [0.05/(78 pairwise interactions of 13 SNPs*7 phenotype domains)].

Experimental studies of B4GALNT2

Constructs: The expression construct pcDNA3.1D/V5-His-TOPO-Beta-1,4-N-Acetyl-Galactosaminyltransferase 2 (B4GALNT2) wt vector (short isoform) was already described (19) and it was kindly provided by Fabio Dall'Olio (University of Bologna, Bologna, Italy). pcDNA3.1D/V5-His-TOPO-B4GALNT2 C406R was generated by site-directed mutagenesis PCR according to the manufacturer's protocol (QuikChange Lightning Site-Directed mutagenesis kit, Agilent Technologies). The primers used to generate C406R mutation are:

Fwd_C406R_: 5'-ggcttccccagccgcgtggtgacca-3'

Rev_C406R_: 5'-tggtcaccacgcggctggggaagcc-3'

Cell lines and culture conditions: Madin-Darby Canine Kidney (MDCK) cells (ATCC_CCL34) were grown in DMEM supplemented with 10% fetal bovine serum (Euroclone), 200 U/ml penicillin, 200 µg/ml streptomycin, and 2 mM glutamine at 37 °C with 5% CO_2. They were transfected using Lipofectamine 2000 (Life Technologies). Selection of stably transfected MDCK cells with 500 µg/ml Geneticin (G418, Life Technologies) was started 48 h after transfection and was maintained for 2 weeks. For immunoreactivity testing experiments, MDCK cells were transduced with a lentiviral vector expressing HA-tagged uromodulin (20) and GFP under the control of the hPGK synthetic bidirectional promoter (21). Transduced cells were isolated through GFP-based cell sorting using a BD FACSAria Fusion (BD Biosciences). Bulk sorted population was stably transfected with the wt B4GALNT2 expressing construct and single cell clones were derived through serial dilution. Clones were characterized for

B4GALNT2 and uromodulin expression; clones B9-C11 (B4GALNT2+) and B11-A11 (B4GALNT2-) were selected on the bases of robust, comparable UMOD expression level.

Protein extracts and western blot analysis: MDCK cells were lysed in lysis buffer (50 mM Tris-HCl, pH 7.4, 150 mM NaCl, 60 mM octyl-β-D-glucopyranoside and 0.1% protease inhibitor cocktail). When needed, samples were N-deglycosylated by incubation with Endoglycosidase H (Endo H) or Peptide:N-glycosidase F (PNGase F) following manufacturer's protocol (New England Biolabs). Lysates were quantified with the Bio-Rad Protein Assay (Bio-Rad). Protein lysates were separated on reducing 8% SDS-PAGE gel, with the exception of immunoreactivity testing, which was carried out in non-reducing conditions, and transferred onto nitrocellulose membrane (GE Healthcare). Western blotting (WB) was performed following standard procedures. Densitometry of signals was carried out by using ImageJ software (22). Possible differences between groups were assessed by non-parametric Mann-Whitney test.

C57BL/6N wild-type mice were housed in a light- and temperature-controlled environment with *ad libitum* access to tap water and standard chow. Mice were sacrificed at 4 weeks by cervical dislocation following anaesthesia with isoflurane (Minrad International Inc.) for kidney collection. Nephron segment microdissection was performed as previously described (23).

RNA isolation and quantitative RT-PCR*:* total RNA was extracted micro-dissected mouse nephron segments using the RNAqueous® kit (Invitrogen). One microgram of RNA was used to perform the reverse transcriptase reaction with iScript TM cDNA Synthesis Kit (Bio-Rad). Changes in target genes' mRNA levels were determined by relative RT-qPCR with a CFX96^{TM} Real-Time PCR Detection System (Bio-Rad) using iQ^{TM} SYBR Green Supermix (Bio-Rad). The

analyses were performed in duplicate with 100nM of both sense and anti-sense primers in a final volume of 20µL using iQ™ SYBR Green Supermix (Bio-Rad). Specific primers were designed using Primer3. PCR conditions were 95°C for 3 min, followed by 40 cycles of 15 sec at 95°C, 30 sec at 60°C. The PCR products were sequenced with the BigDye terminator kit (PerkinElmer Applied Biosystems) using an ABI3100 capillary sequencer (PerkinElmer Applied Biosystems).

Immunofluorescence analysis: Immunofluorescence (IF) of cells was carried out as follows: cells were fixed 20 min in 4% paraformaldehyde (VWR International) and when required permeabilized for 20 min at RT with PBS-Triton 0.5%. Cells were incubated ON at 4°C with primary antibody and subsequently with the appropriate AlexaFluor-labeled secondary antibody (1:500, Life Technologies). Nuclei were stained with 4',6-diamidino-2-phenylindole (DAPI, Life Technologies) and slides were mounted using fluorescence mounting medium (Dako, Agilent Technologies). Slides were visualized under a DM 5000B fluorescence upright microscope (Leica Microsystems GmbH) or under an UltraVIEW ERS spinning disk confocal microscope (PerkinElmer).

The use of human kidney biopsies has been approved by the UCLouvain Ethical Review Board. Mouse kidneys were fixed overnight at 4 °C in 4% formaldehyde (Sigma-Aldrich), dehydrated and subsequently embedded in paraffin. Paraffin blocks were cut into 5 µm-thick sections, deparaffinized in xylene and re-hydrated in decreasing ethanol concentrations. The sections were incubated with primary antibodies overnight at 4° C. After washing steps, sections were incubated with the appropriate AlexaFluor-labeled secondary antibody (1:400, Life Technologies) for 1 hour at room temperature. After the last washing step, sections were mounted using Prolong Gold Anti-fade reagent containing DAPI (Invitrogen) and viewed under

a confocal microscope (Leica Microsystems GmbH) using a ×63 1.4 NA oil immersion objective.

Antibodies: The following primary antibodies were used: rabbit anti-B4GALNT2 (NBP1-91229, Novus Biologicals; 1:500 for WB and 1:50 or 1:500 for IF); anti-beta-actin-peroxidase conjugated (A3854, Merck; 1:100,000 for WB); mouse anti-HA (MMS-101P, Covance; 1:1000 for WB); sheep anti-uromodulin (K90071C, Meridian Life Science Inc.; 1:400 for IF), mouse anti-GM130 (610823; BD Biosciences; IF 1:200), mouse anti-KDEL (10C3, Enzo Life Sciences; IF 1:200); goat anti-AQP2 (sc-9880, Santa Cruz Biotechnology; 1:400 for IF). Mouse anti UMOD-capture (clone T112A12, 1:500 for WB) and mouse anti UMOD-detector (clone T5.G, 1:250 for WB) monoclonal antibodies were generated by somatic cell hybridization following Balb/c mice immunization with uromodulin purified from human urine. These antibodies are not commercially available, are part the ELISA assay (EQ 6821-9601, Euroimmun) (24) and were kindly provided by Euroimmun. Sda antigen was detected by incubating fixed cells with Dolichos biflorus Agglutinin Rhodamine (DBA) (RL-1032, Vector Laboratories, Inc.; 1:200).

Supplementary Figures

Supplementary Figure 1: Quantile-quantile (QQ) plot of observed vs. expected p-values. Panel **A** shows the results of the transethnic meta-analysis of GWAS of antibody-based uromodulin levels, and panel **B** shows results of the meta-analysis of GWAS of aptamer-based uromodulin readout. The y-axis on both panels is truncated at 320.

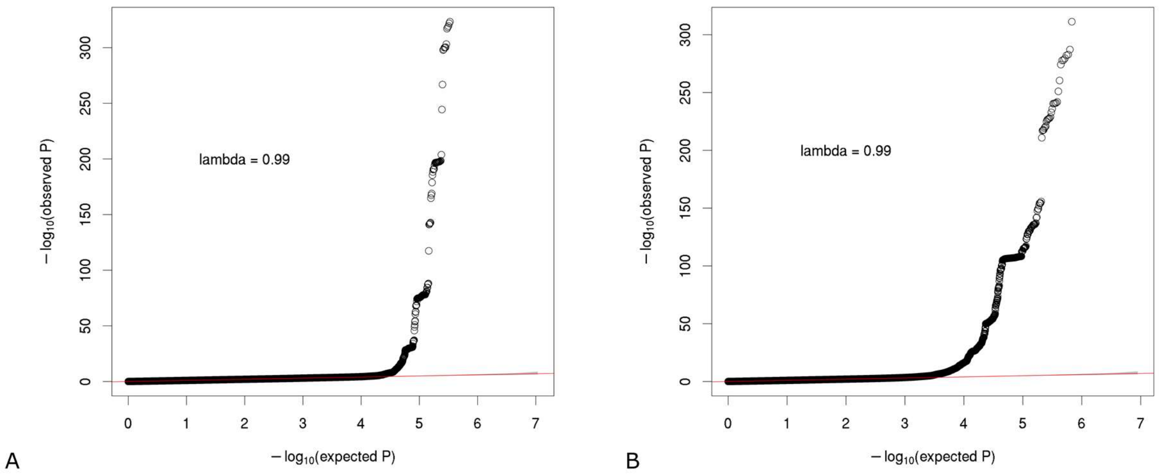

Supplementary Figure 2: Forest plots of the index SNP at each of the three significant loci from the antibody-based primary meta-analysis to illustrate effect sizes in the individual studies and the combined effect estimate. Effect sizes on the x-axis correspond to SD (standard deviation) changes in analyzed uromodulin. The allele to which the depicted effect refers to is shown next to the SNP identifier.

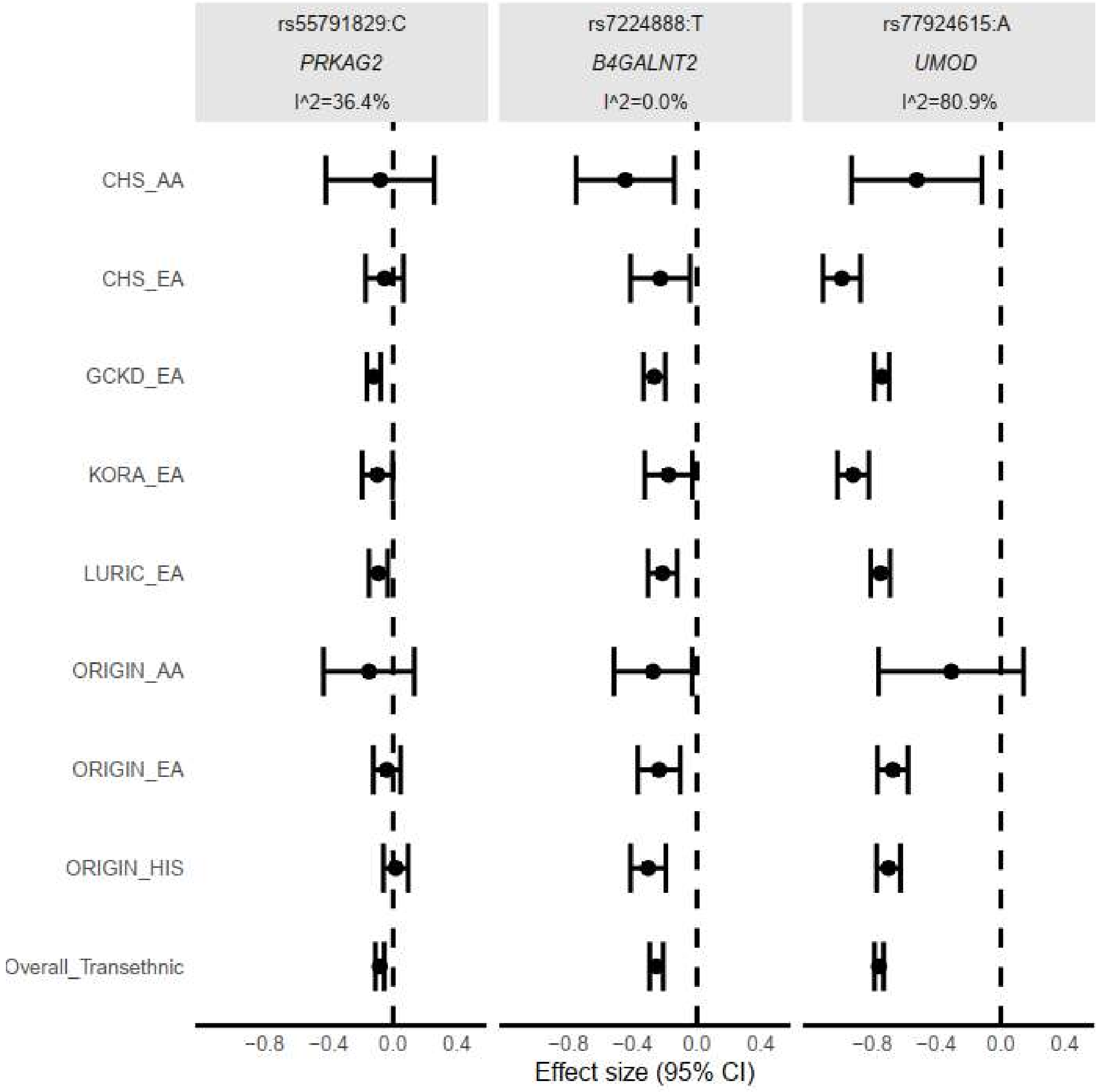

Supplementary Figure 3: Regional association plots of 13 significantly associated loci with uromodulin quantified by both antibody- and aptamer-based assays. The y-axis shows the –$\log_{10}$(two-sided p-value). Correlation (r^2) of each SNP with the index SNP was estimated based on the 1000 Genomes phase 3 EUR reference samples. Plots were generated using LocusZoom v1.4 (25). Chromosomal positions (x-axis) refer to GRCh37 coordinates.

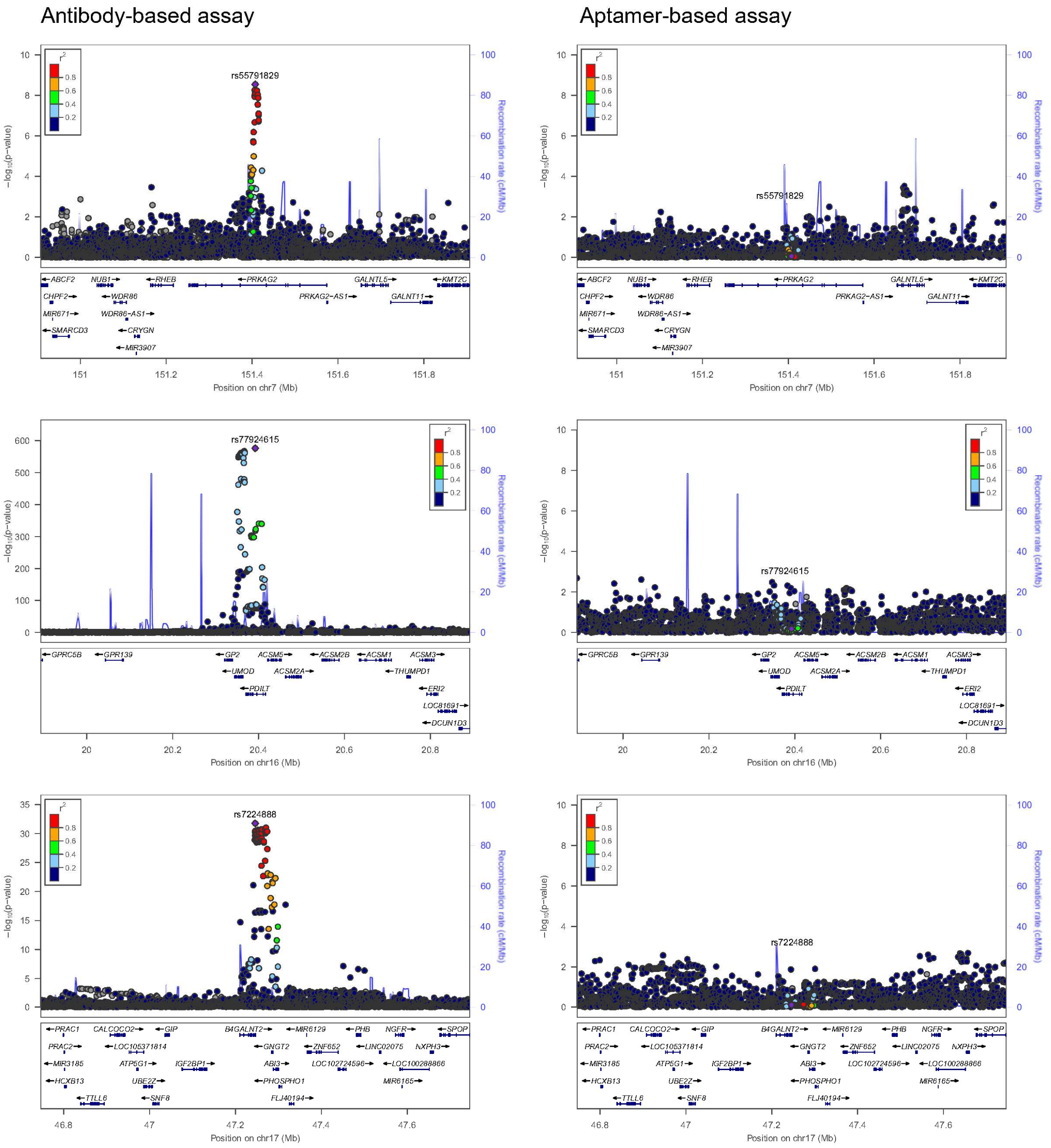

Antibody-based assay   Aptamer-based assay

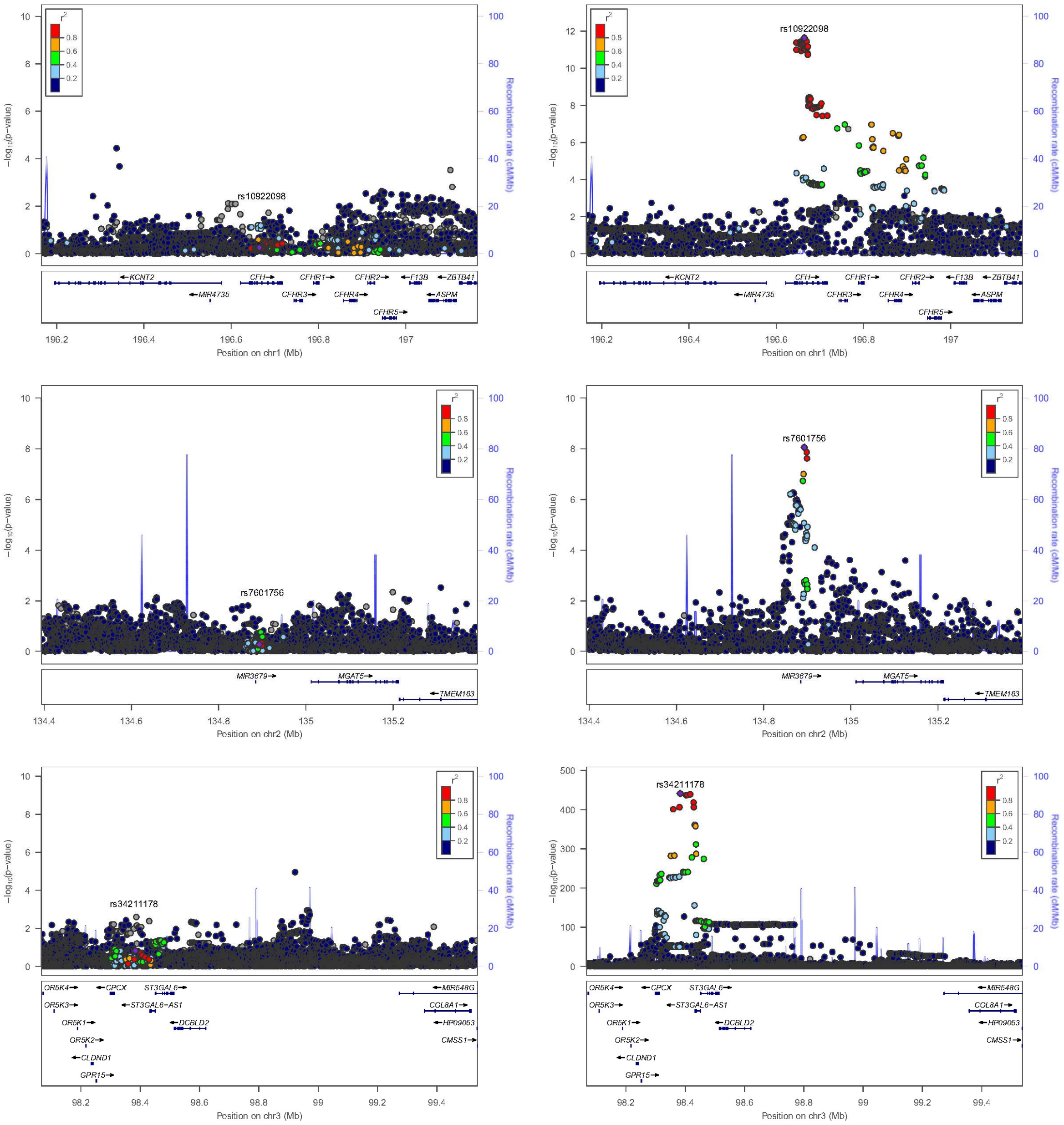

Antibody-based assay

Aptamer-based assay

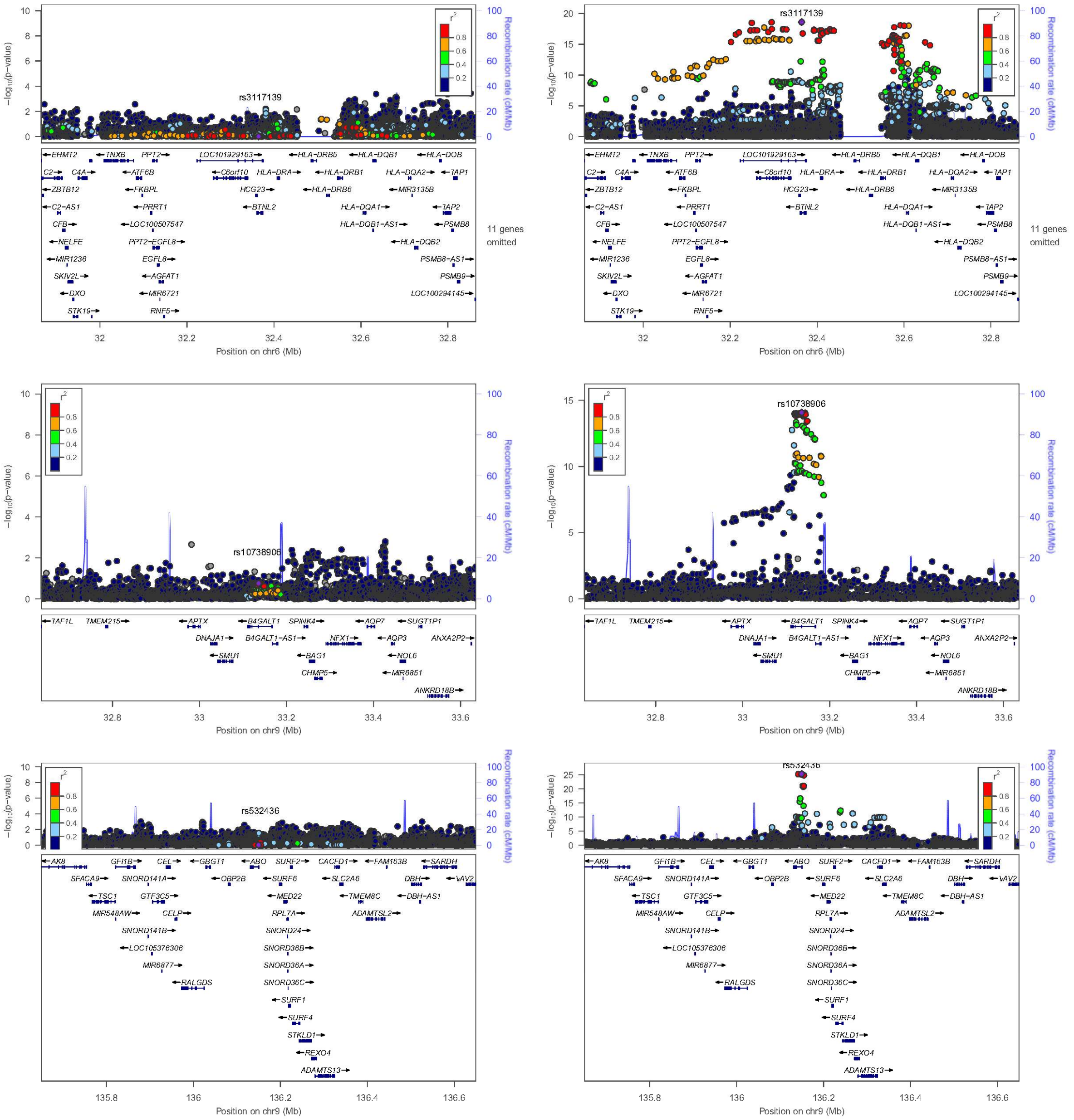

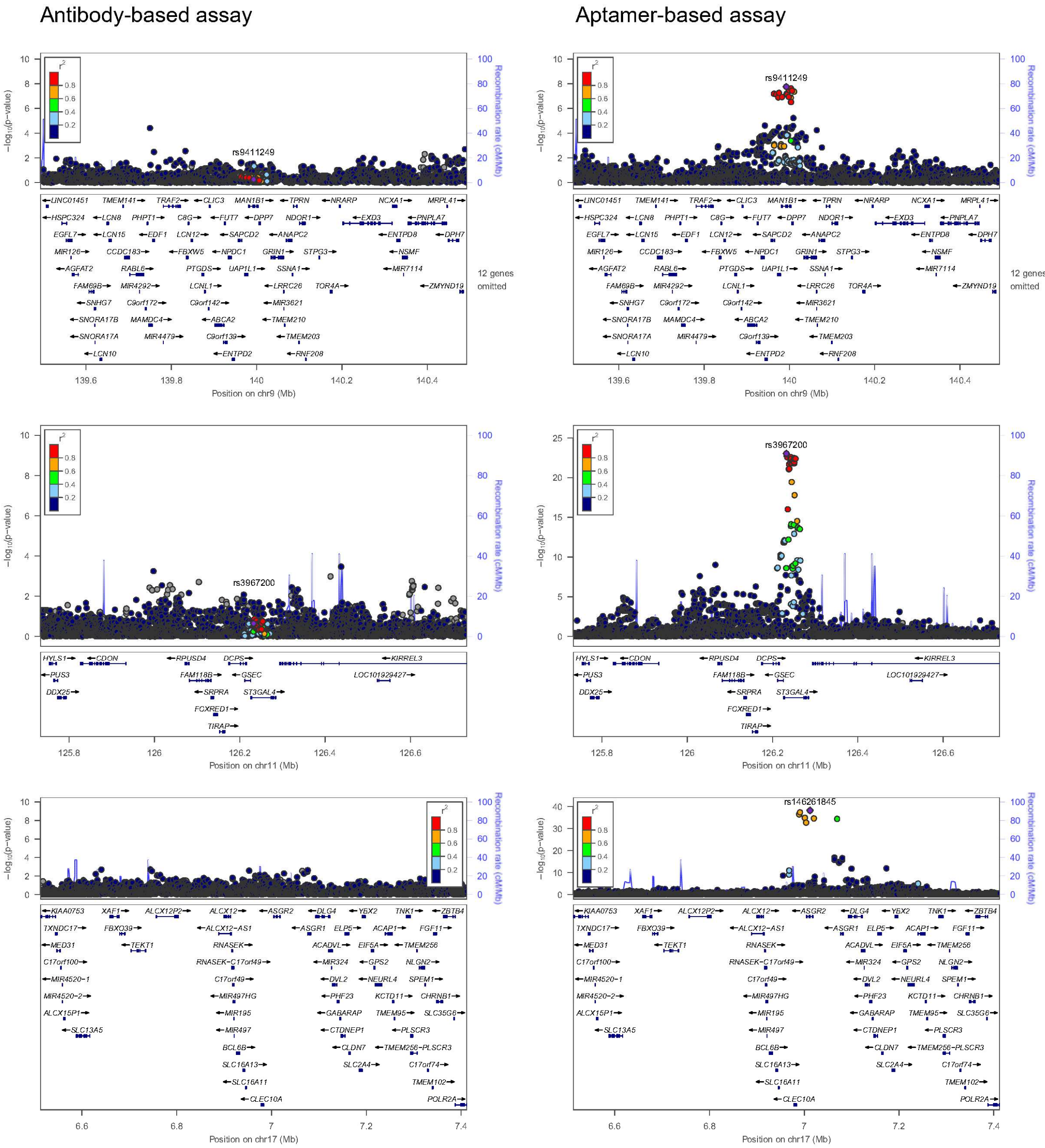

Antibody-based assay
Aptamer-based assay
rs9411249
rs3967200
rs146261845
r²
0.8
0.6
0.4
0.2
−log₁₀(p−value)
Recombination rate (cM/Mb)
12 genes omitted
Position on chr9 (Mb)
Position on chr11 (Mb)
Position on chr17 (Mb)

Antibody-based assay

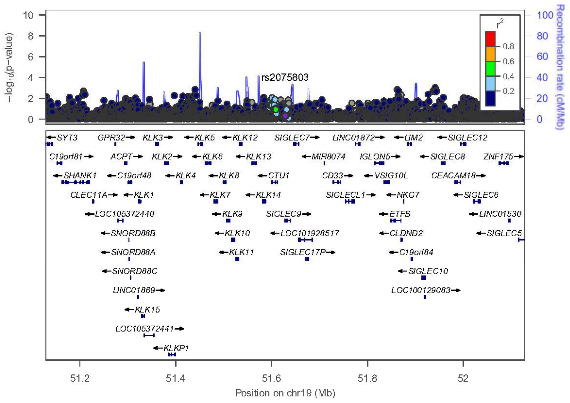

Aptamer-based assay

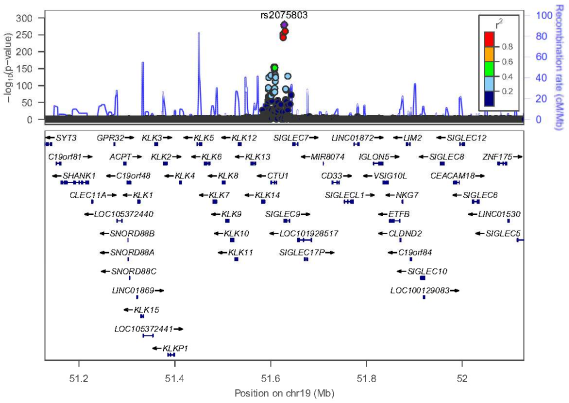

In the regional association plots of rs3117139, the 11 omitted genes are: *C4B_2, C4B, CYP21A2, CYP21A1P, TNXA, RNF5P1, MIR6833, AGER, PBX2, GPSM3, NOTCH4*.
In the regional association plots of rs9411249, the 12 omitted genes are: *LCN6, LOC100128593, MIR6722, CCDC183−AS1, MAN1B1−AS1, CYSRT1, RNF224, SLC34A3, TUBB4B, FAM166A, STPG3−AS1, NELFB*.
In the antibody-based regional association plot on chromosome 17, rs146261845 is not available because it has an MAF smaller than 0.01 and was therefore filtered out.

Supplementary Figure 4: Comparison of genetic effect sizes between the primary, eGFR-adjusted association analyses (x-axis) and a sensitivity-analysis not adjusted for eGFR (y-axis). Dots represent SNPs present in both analyses and with p-value <1e-5. Panel **A** shows results from the antibody-based analyses (513 SNPs), and panel **B** results from the aptamer-based analyses (3244 SNPs). Rho, Spearman's correlation coefficient.

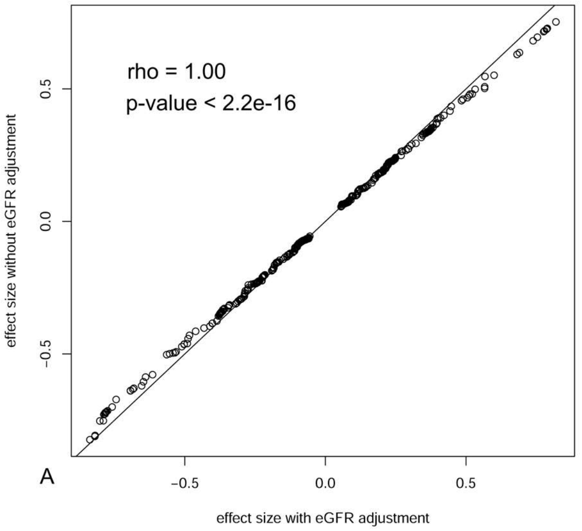

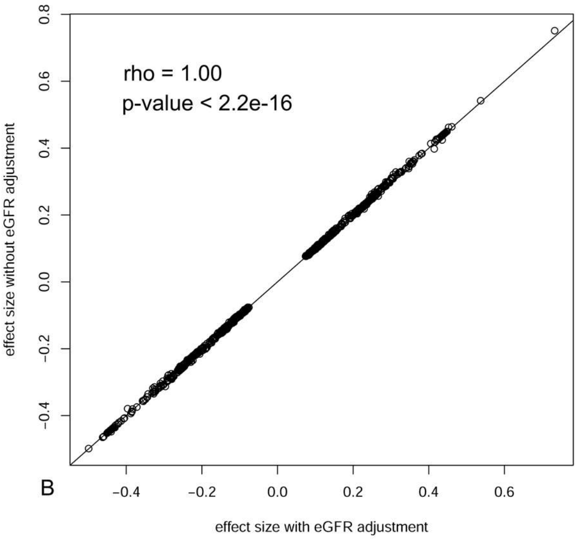

Supplementary Figure 5: Miami plots of sex-stratified primary association analyses of circulating uromodulin. The upper plot shows results from the antibody-based analyses (8,397 men, upwards direction; 5,515 women, downwards direction). The lower plot shows results from the aptamer-based analyses (3,535 men, upwards direction; 3,860 women, downwards direction). The x-axis shows genomic location (GRCh37/hg19 coordinates), and the y-axis the -$\log_{10}$(two-sided p-value) for SNP associations with uromodulin levels. Plots were generated using the EasyStrata v8.6 (26). The red line indicates the genome-wide significance threshold (P<5e-8).

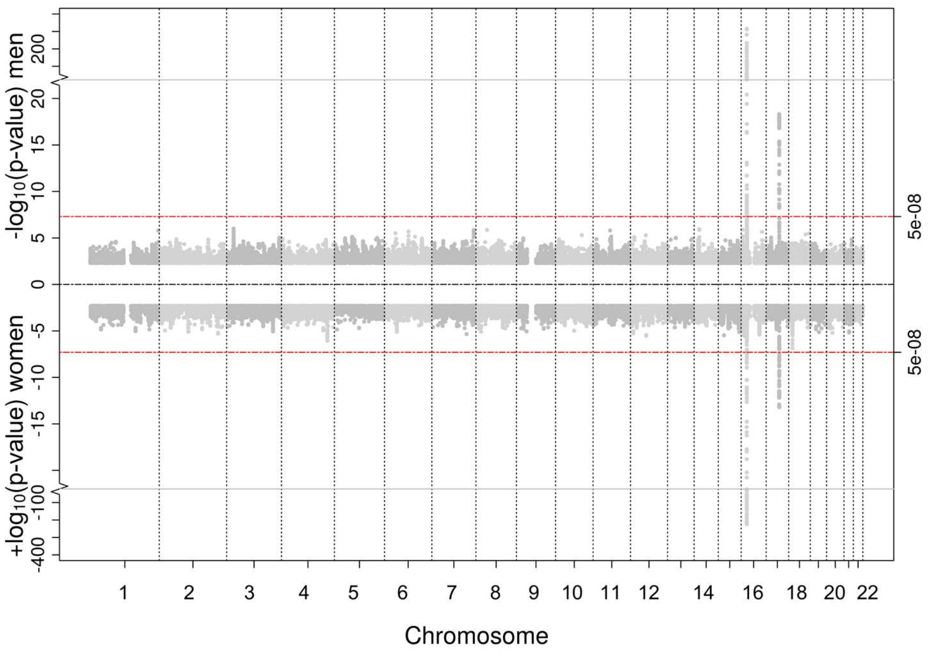

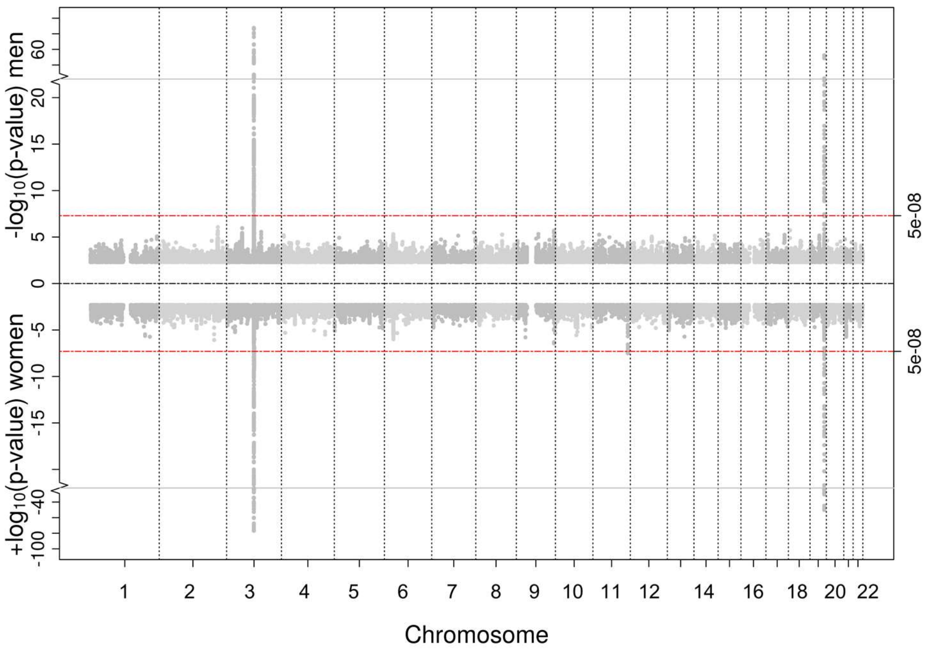

Supplementary Figure 6: Forest plot of the index SNP at each of the 13 significant loci from the primary association analyses to illustrate genetic effect sizes among men, women, and overall. Effect sizes on the x-axis correspond to SD (standard deviation) changes in analyzed uromodulin. The allele to which the depicted effect refers to is shown next to the SNP identifier.

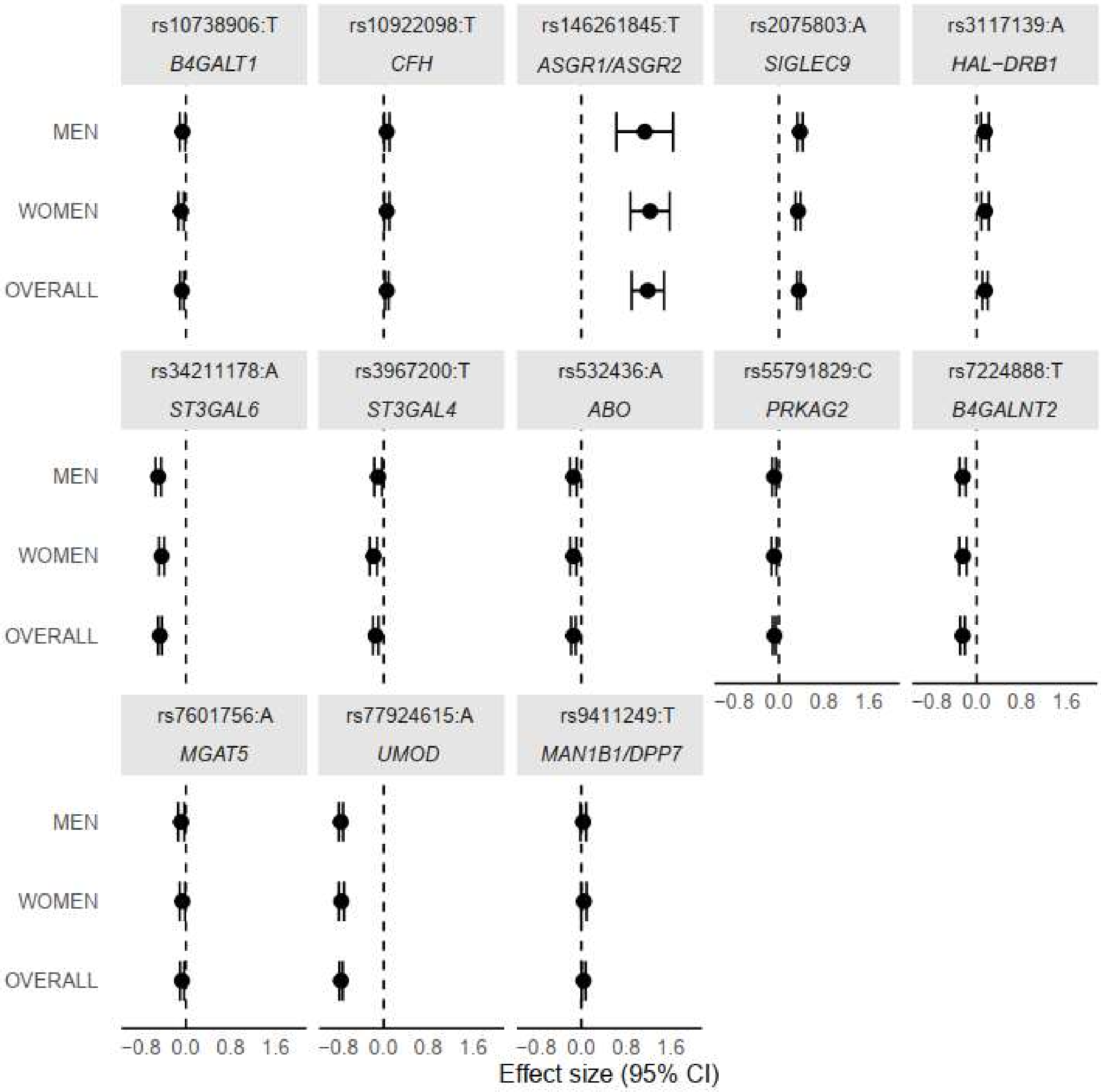

Supplementary Figure 7: Summary of findings from colocalization of uromodulin signals with associations from GWAS of gene expression and protein levels.

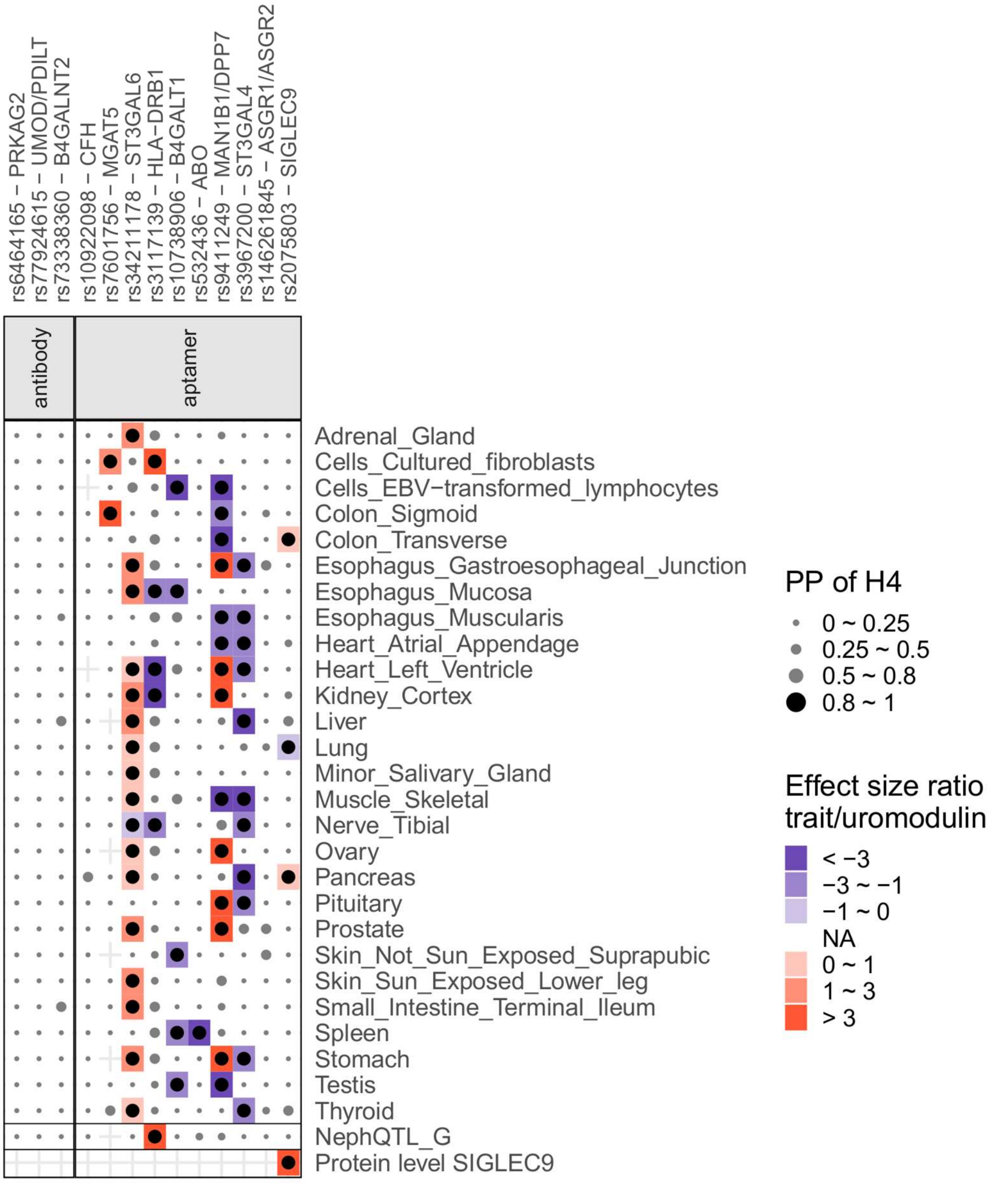

The x-axis indicates the index SNPs with the likely causal genes. The y-axis shows the analyzed eQTL tissues for GTEx and NephQTL. Within each category, horizontal lines separate different data sources (Methods). For pQTL, included proteins had at least one positive colocalization signal (PP of H4>0.8, Methods); for GTEx and NephQTL, included tissues had at least one positive colocalization signal with the expression of the prioritized likely causal gene in *cis*. Dots are black when PP of H4>0.8 and grey otherwise, and scaled in size to reflect the different ranges of the PP of H4. The trait-to-uromodulin effect size ratios are shown as gradient background for positive colocalization signals, with red indicating positive and blue negative changes per unit higher uromodulin levels. H4: hypothesis that one shared SNP underlies the association with two traits; PP: posterior probability.

Supplementary References

1. Joseph CB, Mariniello M, Yoshifuji A, Schiano G, Lake J, Marten J, et al. Meta-GWAS Reveals Novel Genetic Variants Associated with Urinary Excretion of Uromodulin. *J Am Soc Nephrol.* 2022;33(3):511-29.
2. Youhanna S, Weber J, Beaujean V, Glaudemans B, Sobek J, and Devuyst O. Determination of uromodulin in human urine: influence of storage and processing. *Nephrol Dial Transplant.* 2014;29(1):136-45.
3. Arnold M, Raffler J, Pfeufer A, Suhre K, and Kastenmuller G. SNiPA: an interactive, genetic variant-centered annotation browser. *Bioinformatics.* 2015;31(8):1334-6.
4. Schlosser P, Li Y, Sekula P, Raffler J, Grundner-Culemann F, Pietzner M, et al. Genetic studies of urinary metabolites illuminate mechanisms of detoxification and excretion in humans. *Nat Genet.* 2020;52(2):167-76.
5. Yu G, Wang LG, Han Y, and He QY. clusterProfiler: an R package for comparing biological themes among gene clusters. *OMICS.* 2012;16(5):284-7.
6. Dobin A, Davis CA, Schlesinger F, Drenkow J, Zaleski C, Jha S, et al. STAR: ultrafast universal RNA-seq aligner. *Bioinformatics.* 2013;29(1):15-21.
7. Liao Y, Smyth GK, and Shi W. featureCounts: an efficient general purpose program for assigning sequence reads to genomic features. *Bioinformatics.* 2014;30(7):923-30.
8. Sheng X, Guan Y, Ma Z, Wu J, Liu H, Qiu C, et al. Mapping the genetic architecture of human traits to cell types in the kidney identifies mechanisms of disease and potential treatments. *Nat Genet.* 2021;53(9):1322-33.
9. Yang J, Ferreira T, Morris AP, Medland SE, Genetic Investigation of ATC, Replication DIG, et al. Conditional and joint multiple-SNP analysis of GWAS summary statistics identifies additional variants influencing complex traits. *Nat Genet.* 2012;44(4):369-75, S1-3.
10. Wuttke M, Li Y, Li M, Sieber KB, Feitosa MF, Gorski M, et al. A catalog of genetic loci associated with kidney function from analyses of a million individuals. *Nat Genet.* 2019;51(6):957-72.
11. GTEx Consortium. The GTEx Consortium atlas of genetic regulatory effects across human tissues. *Science.* 2020;369(6509):1318-30.
12. Gillies CE, Putler R, Menon R, Otto E, Yasutake K, Nair V, et al. An eQTL Landscape of Kidney Tissue in Human Nephrotic Syndrome. *Am J Hum Genet.* 2018;103(2):232-44.
13. Stanzick KJ, Li Y, Schlosser P, Gorski M, Wuttke M, Thomas LF, et al. Discovery and prioritization of variants and genes for kidney function in >1.2 million individuals. *Nat Commun.* 2021;12(1):4350.
14. Evangelou E, Warren HR, Mosen-Ansorena D, Mifsud B, Pazoki R, Gao H, et al. Genetic analysis of over 1 million people identifies 535 new loci associated with blood pressure traits. *Nat Genet.* 2018;50(10):1412-25.
15. Sun BB, Maranville JC, Peters JE, Stacey D, Staley JR, Blackshaw J, et al. Genomic atlas of the human plasma proteome. *Nature.* 2018;558(7708):73-9.
16. Giambartolomei C, Vukcevic D, Schadt EE, Franke L, Hingorani AD, Wallace C, et al. Bayesian test for colocalisation between pairs of genetic association studies using summary statistics. *PLoS Genet.* 2014;10(5):e1004383.

17. Zheng J, Haberland V, Baird D, Walker V, Haycock PC, Hurle MR, et al. Phenome-wide Mendelian randomization mapping the influence of the plasma proteome on complex diseases. *Nat Genet.* 2020;52(10):1122-31.
18. Sinnott-Armstrong N, Naqvi S, Rivas M, and Pritchard JK. GWAS of three molecular traits highlights core genes and pathways alongside a highly polygenic background. *Elife.* 2021;10:e58615.
19. Malagolini N, Santini D, Chiricolo M, and Dall'Olio F. Biosynthesis and expression of the Sda and sialyl Lewis x antigens in normal and cancer colon. *Glycobiology.* 2007;17(7):688-97.
20. Schaeffer C, Santambrogio S, Perucca S, Casari G, and Rampoldi L. Analysis of uromodulin polymerization provides new insights into the mechanisms regulating ZP domain-mediated protein assembly. *Mol Biol Cell.* 2009;20(2):589-99.
21. Amendola M, Venneri MA, Biffi A, Vigna E, and Naldini L. Coordinate dual-gene transgenesis by lentiviral vectors carrying synthetic bidirectional promoters. *Nat Biotechnol.* 2005;23(1):108-16.
22. Schneider CA, Rasband WS, and Eliceiri KW. NIH Image to ImageJ: 25 years of image analysis. *Nat Methods.* 2012;9(7):671-5.
23. Glaudemans B, Terryn S, Golz N, Brunati M, Cattaneo A, Bachi A, et al. A primary culture system of mouse thick ascending limb cells with preserved function and uromodulin processing. *Pflugers Arch.* 2014;466(2):343-56.
24. Steubl D, Block M, Herbst V, Nockher WA, Schlumberger W, Satanovskij R, et al. Plasma Uromodulin Correlates With Kidney Function and Identifies Early Stages in Chronic Kidney Disease Patients. *Medicine (Baltimore).* 2016;95(10):e3011.
25. Pruim RJ, Welch RP, Sanna S, Teslovich TM, Chines PS, Gliedt TP, et al. LocusZoom: regional visualization of genome-wide association scan results. *Bioinformatics.* 2010;26(18):2336-7.
26. Winkler TW, Kutalik Z, Gorski M, Lottaz C, Kronenberg F, and Heid IM. EasyStrata: evaluation and visualization of stratified genome-wide association meta-analysis data. *Bioinformatics.* 2015;31(2):259-61.
